# Revision of *Eucorydia* Hebard, 1929 from China, with notes on the genus and species worldwide (Blattodea, Corydioidea, Corydiidae)

**DOI:** 10.3897/zookeys.709.14755

**Published:** 2017-10-18

**Authors:** Lu Qiu, Yan-Li Che, Zong-Qing Wang

**Affiliations:** 1 Institute of Entomology, College of Plant Protection, Southwest University, Beibei, Chongqing 400716, P. R. China

**Keywords:** China, Corydiidae, *Eucorydia*, key, new species, new synonym, revision

## Abstract

The cockroach genus *Eucorydia* from China is revised. Five new species are described and illustrated: *Eucorydia linglong* **sp. n.**, *Eucorydia pilosa* **sp. n.**, *Eucorydia splendida* **sp. n.**, *Eucorydia guilinensis* **sp. n.**, and *Eucorydia tangi* **sp. n.**. *Corydia purpuralis* Kirby, 1903 and *Eucorydia paucipilosa* Woo, Guo & Feng, 1986 are confirmed to be junior synonyms of *Eucorydia dasytoides* (Walker, 1868). *Eucorydia hilaris* (Kirby, 1903) is newly recorded from China. This genus and currently known species from around the world are discussed. The status of *Eucorydia maxwelli* (Hanitsch, 1915) is revived. *Corydia plagiata* Walker, 1868 is confirmed to be a junior synonym of *Eucorydia ornata* (Saussure, 1864). A checklist, key, and photographs of members of this genus are provided.

## Introduction

*Eucorydia* is one of the most remarkable genera in Blattodea for its attractive appearance. Most species of this genus are characterized by a shining metallic coloration and bright orange markings. At first, species in this genus were treated as members of *Corydia* Serville. Hebard (1929) erected the genus *Eucorydia*, and included 12 species, dividing them into five groups. Before this study, based on the catalogue of Princis (1963) and latter additional species from China and Japan (Asahina 1971; Woo et al. 1986; Woo and Feng 1988), 16 species were included in this genus, five of which were distributed in China.

*Eucorydia* species are difficult to collect (pers. obs.) and rarely seen in entomological collections (pers. obs.). Because of lack of specimens, this genus has been poorly studied, and in particular lacks descriptions of the male genitalia. Asahina (1971) made limited observations of the male genitalia; but because the genitalia were not dissected from the body, some detailed characters were missed. This article revises this genus from China, studies the male genitalia, and enriches our knowledge of the species in this genus worldwide, providing a solid foundation for future studies.

## Materials and methods

Specimens examined during this research are deposited in the following collections:

**ANSP** The Academy of Natural Sciences of Drexel University, Philadelphia, United States

**NHM** Natural History Museum, London, United Kingdom

**BJFU** Museum of Beijing Forestry University, Beijing, China

**GMNH** Museum of Natural History, Geneva, Switzerland

**GZU** Guizhou University, Guiyang, China

**IZCAS** Institute of Zoology, Chinese Academy of Sciences, Beijing, China

**MHBU** Museum of Hebei University, Baoding, China

**NHMV** Natural History Museum Vienna, Vienna, Austria

**OUM** Oxford University Museum of Natural History, Oxford, UK

**SHNU** Shanghai Normal University, Shanghai, China

**SWU** Institute of Entomology, Southwest University, Chongqing, China

**CHZC** Cheng-Hui Zhan Personal Collection, Guangdong, China

**LQCC** Lu Qiu Personal Collection, Sichuan, China

**JZZC** Jia-Zhi Zhang Personal Collection, Shanghai, China

Morphological terminology used in this paper mainly follows Roth (2003), genitalia terms mainly follow Klass (1997), and venation terms mainly follow Kukalová-Peck and Lawrence (2004) with the modification by Li and Wang (2015).

The genital segments of the examined specimens were macerated in 10% NaOH and observed in glycerin jelly using a Motic K400 stereomicroscope and a Leica® M205A stereomicroscope. All drawings were made with the aid of Adobe Photoshop® CS5, a Leica® M205A stereomicroscope and a Motic® K400 stereomicroscope. Photographs of the specimens were made using a Canon® 50D plus a Canon® EF 100mm f/2.8L IS USM Macro lens combined with Helicon Focus® software. Habitus photos were taken using a Nikon® Coolpix P7700 digital camera. Living female and ootheca pictures were taken using a Canon® 50D plus a Canon® EF 100mm f/2.8L IS USM Macro lens. Photos of other characters were taken using a Leica® M205A stereomicroscope. All photographs mentioned above were modified in Adobe Photoshop® CS5.

## Result

### Genus Eucorydia

Taxon classificationAnimaliaBlattodeaCorydiidae

Hebard, 1929

Eucorydia Hebard, 1929: 96; Princis 1963: 81; Asahina 1971: 256.

#### Type species.

*Eucorydia westwoodi* (Gerstaecker, 1861)

#### Diagnosis.

This genus is remarkable for its brilliant coloration; most species are a shiny metallic blue or green, with bright orange coloration on tegmina and abdomen.

#### Description.

**Figure 1.**
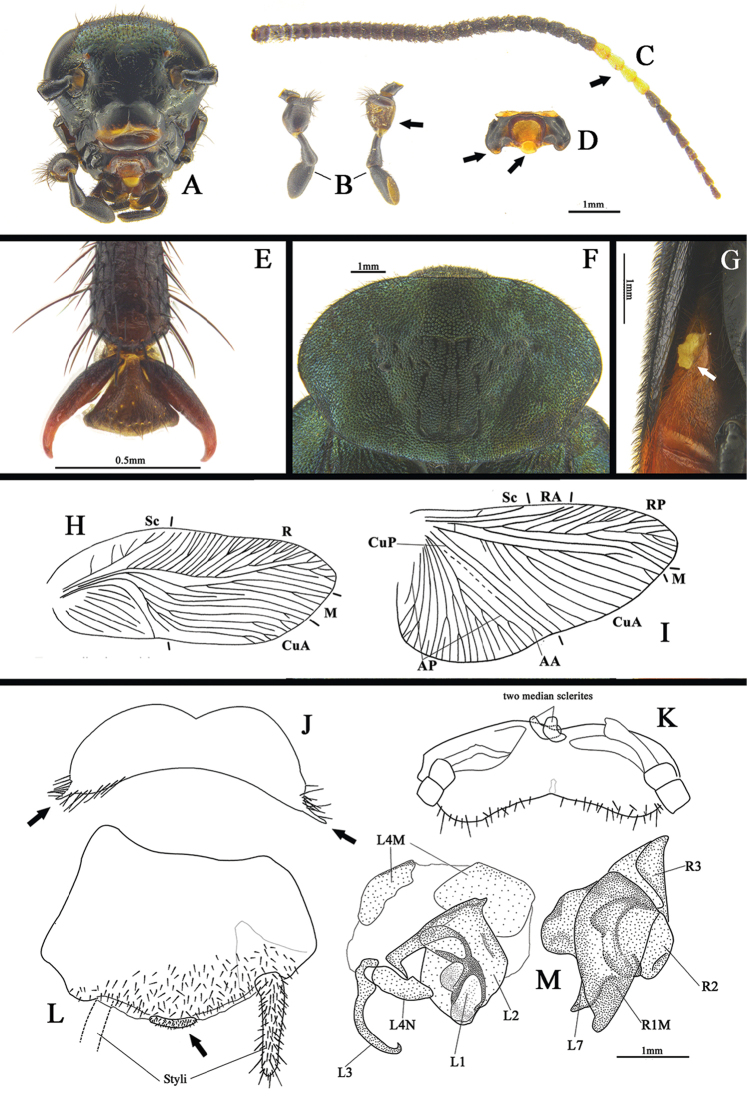
**A–M** Features of male *Eucorydia* Hebard **A** head **B** maxillary palpus **C** antenna **D** labrum **E** arolia **F** pronotum **G** eversible gland on abdomen **H** venation of tegmen **I** venation of wing **J** T8, dorsal **K** supra-anal plate, ventral **L** subgenital plate, ventral **M** genitalia, dorsal.

Male: body length 8.0–18.5 mm, including tegmina 11.0–22.3 mm. Body small, bright, usually metallic greenish blue to blue, some species blackish colored, tegmina usually with orange band, spots or occupied by large orange areas, abdomen occupied by small to large orange areas. The coloration of the pubescence or setae on the body surface usually brown to black, or identical to the coloration where they are inserted (e.g., the yellow band usually with yellow pubescence), but some species may have additional white or gray pubescence in specific areas. **Head**: Roundly triangular (Fig. 1A), usually black with vertex slightly metallic, thickly pubescent, much denser at vertex; eyes wide apart, moderate size, interocular space narrower than the distance between antennal sockets, ocelli very small, reduced to small spots; two shallow dimples between the antennal sockets; antennae with basal segment elongate, the remaining segments short, but thickened medially and then thinner toward apex, two to six segments near the apex white (arrow in Fig. 1C); clypeus small, well divided into ante-clypeus and hind-clypeus, and hind-clypeus also distinctly divided by a longitudinal line medially; labrum specialized, transverse, median with a round impression, hind lateral corners protruded (arrow in Fig. 1D); maxillary palpi with 3^rd^ segment enlarged, concave and pubescent (arrow in Fig. 1B), 4^th^ segment with base thin, distal portion thick, 5^th^ segment with apex truncated, hollow. **Pronotum**: transverse, usually metallic, some species black and with yellowish stripes, surface densely nodulose and setose, disc with many smooth and thin stripes forming a symmetrical marking (Fig. 1F). **Tegmina and wings**: Tegmina broad, exceeding the end of abdomen, metallic or black, usually with orange band, spotted or occupied by large orange areas; wings yellow to dark brown, some species with a transparent stripe across the middle of the wing, RA end usually with an elongate yellow spot. Venation as in Fig. 1H–I (*Eucorydia dasytoides* is an example), tegmen with a simple Sc which only has several small branches, R well-branched and occupying approximately 2/5 of the tegmen, M basally with a bifurcate branch and with approximately three branches distally, CuA branched basally, with approximately four branches. Hind wing with a single RA, RP occupying the distal margin, M single, CuA with approximately eight straight and paralleled branches. **Legs**: Dark brown to black, sometimes slightly metallic, with unequal length setae, apical femur with a spine, tarsal claws symmetrical, arolia present (Fig. 1E). **Abdomen**: Generally two types, one type totally orange except the last apical segments, the other type only with lateral portions orange, the rest of area dark brown to black, and slightly metallic; the 8^th^ terga specialized, lateral corners protruded, spinous, elongate (Fig. 1J); eversible glands present (arrow in Fig. 1G). Supra-anal plate short, transverse, pubescent, median concave, the shape of hind margin varies, two median sclerites present, paraprocts asymmetrical, cerci long (Fig. 1K). Subgenital plate protruded, well setose, apex with a small and well setose isolated area (arrow in Fig. 1L), styli thick, long, setose. **Genitalia**: Left phallomere: L1 with anterior portion elongate, round, hind portion bifurcate, membrane medially, L2 enlarged, plate-like, left elongate, curved, L3 thin, strongly and roundly curved, L4N simple, L4M transparent, membrane, with two sclerotized portions, L7 well-developed, integrated with the right phallomere as an appendage sclerite, generally with two protruded parts, the basal one varied in shape, the distal one usually elongate, apex narrowed. Right phallomere: R1M large, elongate, subtransparent, hind apex protruded, round, R3 small, curved, R2 round, irregular, shape varies (Fig. 1M).

Female: winged, arolia present; generally similar to the male, but differing in the following features: 1) labrum not specialized, apex round, the 3^rd^ segment of maxillary palpus normal, not enlarged and concave; 2) tegmina short in that they are usually reaching to, or only slightly beyond, the end of abdomen; 3) supra-anal plate large, round and protruded, apex slightly emarginated, median with a longitudinal line, cerci short; 4) subgenital plate with hind portion protruded, bulging.

Nymph: yellowish brown to dark brown, well pubescent, antennae near the apex white (Fig. 14H).

Ootheca: keel with distinct serrations. The longitudinal line on the surface sharp.

#### Natural history.

Male usually visiting flowers during the day (e.g., *E. dasytoides* was observed visiting the flowers of *Castanopsis carlesii* and *Acer albopurpurascens* in Taiwan, Wen-I Chou, pers. obs.), female can be found hiding under the bark of rotten wood (Yi-Zhou Liu, pers. obs. for *E. linglong* sp. n.). Sometimes the individuals can be found on the ground (Lu Qiu, pers. obs. for *E. dasytoides*; Jin Chen, pers. obs. for *E. linglong* sp. n.), or observed flying on the mountain top (Wen-I Chou and De-Yao Zhou, pers. obs. for *E. dasytoides*).

#### Distribution.

China, India, Myanmar, Vietnam, Japan, Southeast Asia.

#### Key to Eucorydia species worldwide

**Table:** 

1	Pronotum with two yellowish spots	***E. ornata***
–	Pronotum without two yellow spots	**2**
2	Tegmina unicolored	**3**
–	Tegmina not unicolored	**4**
3	Body black (Fig. 12D-E)	***E. tristis***
–	Body metallic green	***E. yasumatsui***
4	Tegmina metallic green to blue, with only two small spots on the lateral margins	**5**
–	Tegmina brown, dull blue or metallic green to blue, pattern not as above	**9**
5	Tegmina with whitish pubescence on the metallic area	**6**
–	Tegmina without white pubescence on metallic area, abdomen orange except for the last three sternites (Fig. 12A–B)	***E. coerulea***
6	Body very small, less than 10 mm (without tegmina) both sexes, terga strongly metallic purplish (Fig. 11L–M)	***E. gemma***
–	Body not very small, more than 10 mm (without tegmina) both sexes	**7**
7	White pubescence on tegmina circle-shaped	**8**
–	White pubescence on tegmina band-shaped (Fig. 7L, N)	***E. linglong* sp. n.**
8	Yellow spots on tegmina elongate (Fig. 11A, D)	***E. aenea***
–	Yellow spots on tegmina small (Fig. 11H, K)	***E. forceps***
9	Tegmina with distal half totally orange, basal half metallic green to blue; abdomen orange except the last two to four sternites	**10**
–	Not as above	**12**
10	Tegmina with anal areas half orange, half metallic (Fig. 9A)	***E. xizangensis***
–	Tegmina with anal areas totally metallic	**11**
11	Border between the metallic and orange areas W-shaped (Fig. 9G, I)	***E. splendida* sp. n.**
–	Border between the metallic and orange area slightly waved (Fig. 14L–M)	***E. hilaris***
12	Pronotum metallic green with yellowish pubescence; tegmina orange, base metallic green, median and distal with four irregular spots on each tegmen (Fig. 12G)	***E. multimaculata***
–	Not as above	**13**
13	Head orange	**male *E. maxwelli***
–	Head dark-colored	**14**
14	Tegmina orange, apex brown, basal lateral margins brown, extending inward and enlarged, anal areas totally orange (Fig. 13H)	**female *E. maxwelli***
–	Not as above	**15**
15	Body dull blue, tegmina with a large spot medially on the sutural margin	***E. westwoodi***
–	Not as above	**16**
16	Tegmina with white or yellowish white pubescence on metallic area	**17**
–	Tegmina without white or yellowish white pubescence on metallic area	**19**
17	Body large, male body more than 18 mm (including tegmina); tegmina base with whitish pubescence, pronotum without white pubescence (Fig. 7F)	***E. pilosa* sp. n.**
–	Body small, male body less than 17 mm(including tegmina), both tegmina base and pronotum with white or yellowish white pubescence	**18**
18	Body broad and robust, male body length about 16 mm (including tegmina); pubescence on the tegmina white extending along the sutural margin of left tegmen (Fig. 7J)	***E. tangi* sp. n.**
–	Body narrow and short, male body length less than 14.5 mm; pubescence on tegmina and pronotum yellowish white (Fig. 7H)	***E. guilinensis* sp. n.**
19	Body small, male body only 15 mm (including tegmina)	***E. yunnanensis***
–	Body large, male body more than 18 mm (including tegmina)	***E. dasytoides***

#### Checklist of *Eucorydia* worldwide (Chinese species in bold)

*Eucorydia aenea* (Brunner von Wattenwyl, 1865)—India, Thailand, Vietnam, Myanmar

*Eucorydia coerulea* (Shelford, 1906)—Malaysia

***Eucorydia dasytoides* (Walker, 1868)**—China, Vietnam

*Eucorydia forceps* (Hanitsch, 1915)—Malaysia

*Eucorydia gemma* Hebard, 1929—Indonesia

***Eucorydia guilinensis* sp. n.**—China

***Eucorydia hilaris* (Kirby, 1903)**—China

***Eucorydia linglong* sp. n.**—China, Vietnam

*Eucorydia maxwelli* (Hanitsch, 1915)—Malay Peninsula, Sumatra and Borneo

*Eucorydia multimaculata* Bruijning, 1948—Indonesia

*Eucorydia ornata* (Saussure, 1864)—India, Myanmar

***Eucorydia pilosa* sp. n.**—China

***Eucorydia splendida* sp. n.**—China

***Eucorydia tangi* sp. n.**—China

*Eucorydia tristis* Hanitsch, 1929—Indonesia

*Eucorydia westwoodi* (Gerstaecker, 1861)—India, Nepal

***Eucorydia xizangensis* Woo & Feng, 1988**—China

*Eucorydia yasumatsui* Asahina, 1971—Japan

***Eucorydia yunnanensis* Woo, Guo & Feng, 1986**—China

*Eucorydia* sp. 1—Thailand

***Eucorydia* sp. 2**—China

### Species found in China

#### Eucorydia dasytoides

Taxon classificationAnimaliaBlattodeaCorydiidae

(Walker, 1868)

**Figure 2.**
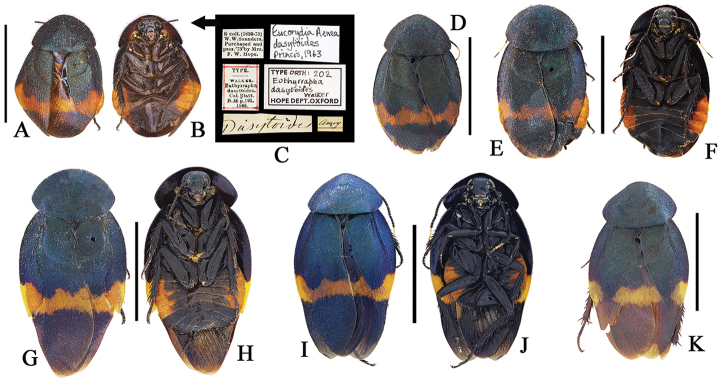
**A–K** *Eucorydia dasytoides*, the type population **A–C** holotype of *Euthyrrhapha dasytoides*: **A–B** habitus, female **C** label [A–C photographed by Katherine Child and provided by Amoret Spooner, copyright Oxford University Museum of Natural History, Oxford (OUM)] **D** female, from Huaihua, Hunan **E–F** female, from Mt. Fanjingshan, Guizhou **G–H** male, from Mt. Hupingshan, Hunan **I–J** male, from Zhenyuan, Guizhou **K** male, from Yueyang, Hunan. Scale bars 10 mm.

**Figure 3.**
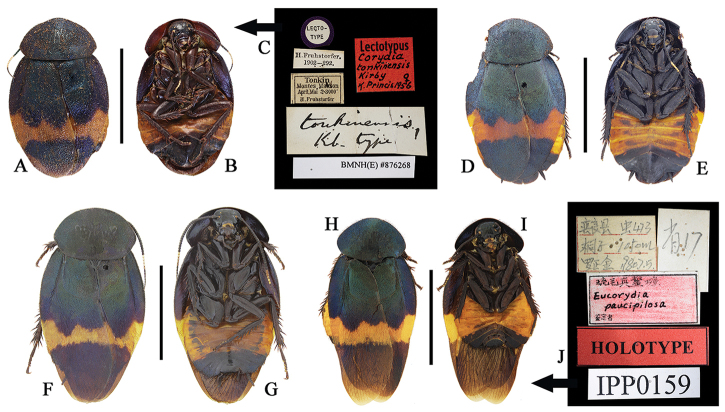
**A–J** *Eucorydia dasytoides*, the *tonkinensis* population **A–C** lectotype of *Corydia tonkinensis*: **A–B** habitus, female **C** label [A–C photographed by Zong-Qing Wang, copyright The Natural History Museum, United Kingdom (NHM)] **D–E** female, from Jinxiu, Guangxi **F–G** male, from Jinxiu, Guangxi **H–J** holotype of *Eucorydia paucipilosa*: **H–I** habitus, male **J** label. Scale bars 10 mm.

**Figure 4.**
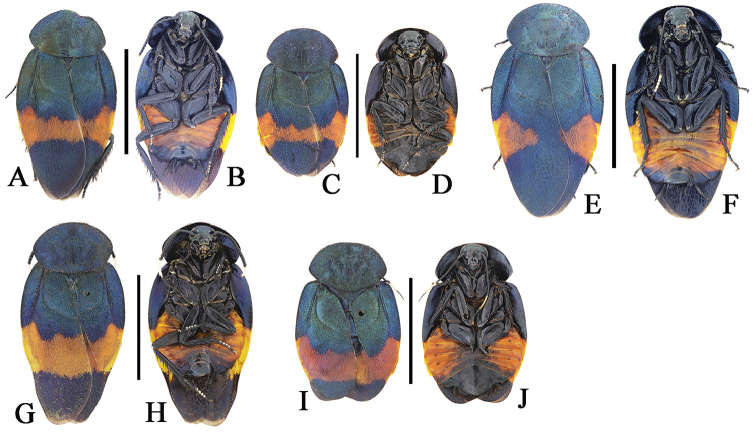
**A–J** *Eucorydia dasytoides* from Hainan and Taiwan **A–D** the *zonata* population from Taoyuan, Taiwan: **A–B** male **C–D** female **E–F** the *taitoensis* population, male, from Taidong, Taiwan **G–J** the Hainan population from Mt. Jianfengling: **G–H** male **I–J** female. Scale bars 10 mm.

**Figure 5.**
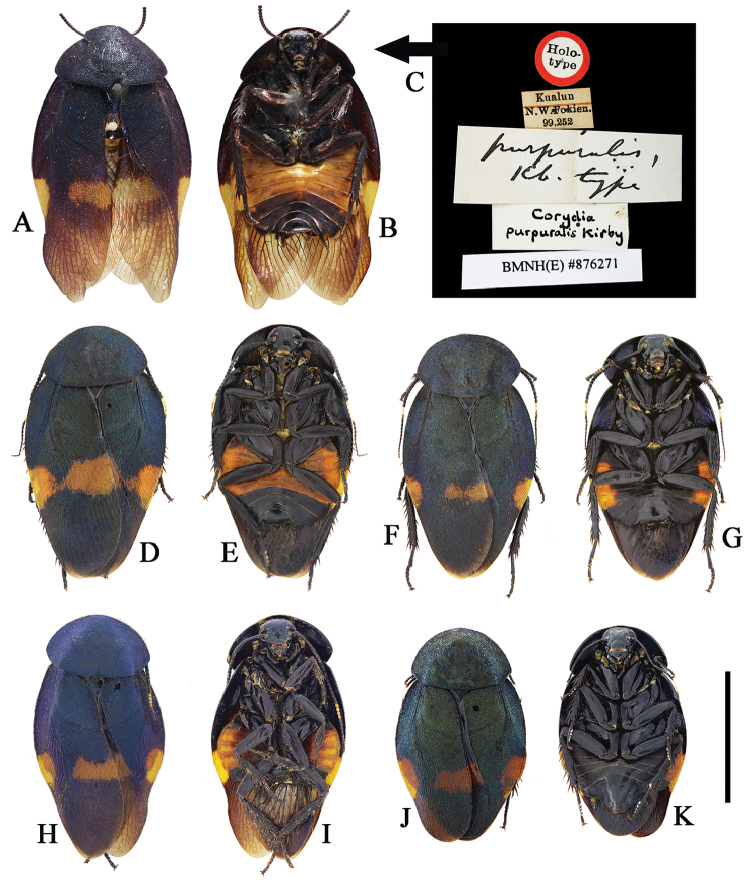
**A–K** Eucorydia dasytoides, the purpuralis population **A–C** holotype of Corydia purpuralis: **A–B** habitus, male **C** label [**A–C** photographed by Zong-Qing Wang, copyright The Natural History Museum, United Kingdom (NHM)] **D–E** male, from Mt. Wuyishan, Fujian **F–I** males, from Mt. Tianmushan, Zhejiang **J–K** female, from Mt. Dapanshan, Zhejiang. Scale bar 10 mm.

**Figure 6.**
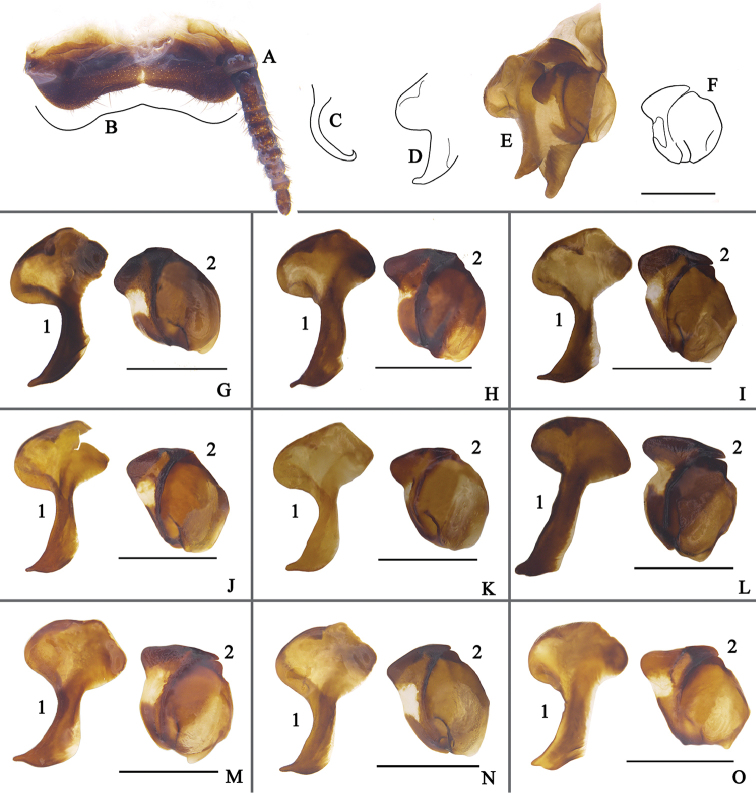
**A–O** Features of male *Eucorydia dasytoides* **A** supra-anal plate, ventral **B** hind margin of the supra-anal plate **C** genital hook (L3) **D** appendage sclerite (L7) **E** right phallomere with L7 **F** R2 **G–O** variation of the appendage sclerite (**1**) and R2 (**2**) from different localities: **G** from Jinxiu, Guangxi **H** from Mt. Jiangfengling, Hainan **I** from Taoyuan, Taiwan **J** from Taidong, Taiwan **K** from Mt. Tianmushan, Zhejiang **L** from Mt. Wuyishan, Fujian **M** from Yueyang, Hunan **N** from Zhenyuan, Guizhou **O** from Zhaotong, Yunnan. Scale bars 1 mm.

Figs 2A–K ; 3A–J ; 4A–J ; 5A–K ; 6A–O ; 14E–I

Euthyrrhapha dasytoides Walker, 1868: 191, male (actually female), “Amoy”.
Corydia dasytoides : Kirby 1904: 167; Hanitsch 1927: 41.
Eucorydia dasytoides : Hebard 1929: 98; Wu 1935: 28; Princis 1952: 35; Princis 1957: 90; Princis 1963: 82; Asahina 1971: 259.
Eucorydia aenea dasytoides : Asahina 1971: 262 (Taxonomic considerations).
Corydia tonkinensis Kirby, 1903: 405, 2 females, “Tonkin”.
Eucorydia tonkinensis : Hebard 1929, 97, 1 male, “Chapa, Tonkin, May 8, 1918, by Jeanvoine”.
Corydia purpuralis Kirby, 1903: 405; Kirby 1904: 167; Hanitsch 1927: 41. **Syn. n.**
Eucorydia purpuralis : Hebard 1929: 98; Wu 1935: 28; Princis 1952: 35; Bey-Bienko,1954: 24; Princis 1957: 90; Princis 1963: 82; Woo et al. 1986: 154.
Corydia zonata Shiraki, 1907: 110, male, “Horisha, Taiwan”; Karny, 1915: 62; Hanitsch 1927: 41; Shiraki 1931: 175.
Corydia zonata var. taitoensis Shiraki, 1931: 176.
Eucorydia purpularis var. taitoensis : Princis 1963: 82.
Eucorydia paucipilosa Woo, Guo & Feng, 1986: 156, figs 1–4; Fenget al. 1997: 175, fig. 69a–b. **Syn. n.**

##### Material examined.

**CHINA: Guangxi** (*tonkinensis* population): 1 male (IZCAS), Hongtan Waterfall [红滩瀑布], Huaping Natural Reserve [花坪自然保护区], Longsheng County [龙胜县], Guilin City [桂林市], 900m, 12.VI.1963, Yong-Shan Shi leg.; 1 female (SWU), Mt. Zuohushan [坐虎山], Huaping Natural Reserve, Longsheng County, Guilin City, 9.VI.1963, Ji-Kun Yang leg.; 1 female (IZCAS), Mt. Tiantangshan [天堂山], Zhongliang Township [忠良乡], Jinxiu County [金秀县], Laibin City [来宾市], 600m, 11.V.1999, Xue-Zhong Zhang leg.; 2 males (SWU), Zhongliang Township, Jinxiu County, Laibin City, 1200 m, 20.IV.2016, local collector leg., purchased by Cheng-Hui Zhan. **Hunan** (type population): 1 male (SWU), Mt. Hupingshan [壶瓶山], Shimen County [石门县], Changde City [常德市], VI.1987, no collector recorded; 1 female (SWU), Yuanling County [沅陵县], Huaihua City [怀化市], no more data recorded; 1 female (SWU), Yueyang City [岳阳市], 6.VII.? (no year data), Liu & Wang (only surnames) leg. **Guizhou** (type population): 1 female (IZCAS), Mt. Fanjingshan [梵净山], Jiangkou County [江口县], Tongren City [铜仁市], 12.VII.1988, Shu-Yong Wang leg.; 1 female (SWU, ex YSLC), Tiexi [铁溪], Zhenyuan County [镇远县], 25.V.2016, Yong-Shan Guo & Shu-Lin Yang leg. 5 males and 1 female (GZU), Daheba [大河坝], Yanhe County [沿河县], 450–700m, 5–12.VI.2007, Qiong-Zhang Song leg. **Fujian** (*purpuralis* population): 1 male (SWU, conserved in 100% alcohol), Guadun [挂墩], Tongmu Village [桐木村], Xingcun Town [星村镇], Wuyishan City [武夷山市], 9.VII.2013, Shun-Hua Gui leg.; 1 male (SWU), Guadun, Tongmu Village, Xingcun Town, Wuyishan City, 1227m, 12.VII.2009, Jian-Yue Qiu leg.; 1 male (SWU), Chong’an Town (now Chong’an Street), Wuyishan City, ?.VII.1987, Dun-Qing Wang leg.; 1 male (IZCAS), Guadun, Tongmu Village, Xingcun Town, Wuyishan City, 900–1160m, 7.VII.1963, You-Wei Zhang leg.; 1 male (BJFU), “Guadang (Guadun)”, Mt. Wuyishan, Wuyishan City, 28.VI.1981, no collector recorded; 1 male (IZCAS), Qiliqiao [七里桥], Xingcun Town, Wuyishan City, 840m, 12.VII.1963, You-Wei Zhang leg.; 1 male (SWU), Huangxizhou [黄溪州], Mt. Wuyishan, Wuyishan City, 27.V.2004, Cai-Xia Yuan & Jing Li leg.; 1 male (SWU, ex SNU), Wanmulin [万木林], Jian’ou City [建瓯市], Nanping City [南平市], V.1985, no collector recorded; 1 male (SWU), Kuiqi Village [魁歧村], Mawei Town [马尾镇], Fuzhou City [福州市], 15.VI.1948, no collector recorded. **Zhejiang** (*purpuralis* population): 1 male, 1 female (SWU), “Tienmushan (Mt. Tianmushan [天目山], Lin’an City [临安市]), July 11 1937”, no collector recorded; 1 female (SWU), “Tienmushan (Mt. Tianmushan, Lin’an City), July 7 1936”, no collector recorded; 1 male (SWU), Sanliting Pavilion [三里亭], Mt. Tianmushan, Lin’an City, 27.VI.1957, Fa-Sheng Li leg.; 1 female (SWU), Chanyuansi Temple [禅源寺], Mt. Tianmushan, Lin’an City, 1.VII.1957, Ji-Kun Yang leg.; 2 females (MHBU), Chanyuansi Temple, Mt. Tianmushan, Lin’an City, 19.VII.2014, Sai-Hong Dong & Shan-Shan Liu leg.; 2 males (DYZC), Xianrending Peak [仙人顶], Mt. Xitianmushan, Lin’an City, 1500m, by netting, 17.VII.2014, De-Yao Zhou leg.; 2 males (SWU), “T’ienmu Shan (Mt. Tianmushan, Lin’an City), 24.VI.1936, O. PIEL. coll.”; 1 male (SWU), “T’ienmu Shan (Mt. Tianmushan, Lin’an City), 13.VI.1936, O. PIEL. coll.”; 2 females (SWU), “T’ienmu Shan (Mt. Tianmushan, Lin’an City), 31.VI and 1.VII.1936, O. PIEL. coll.”; 1 female (SWU), “T’ienmu Shan (Mt. Tianmushan, Lin’an City), 17.V.1937”, no collector recorded; 1 male (SWU), abdomen missing, Mt. Tianmushan, Lin’an City, 350m-1100m, 14.VII.1963, Hui-Tai Fang leg.; 1 male (LQCC, ex SNU), Mt. Tianmushan, Lin’an City, VI.1986, no collector recorded; 1 female (LQCC), Mt. Xitianmushan, Lin’an City, 800m, 8.VII.2006, Zhi-Zhou Yu leg.; 1 female (SWU), Mt. Dapanshan [大盘山], Pan’an County [磐安县], Jinhua City [金华市], 500m, 19.VII.2014, Tie-Xiong Zhao leg.; 1 nymph (MHBU), Administration of Mt. Tianmushan, 30.VII.2011, Ji-Bin Liang & Zhen-Xing Zhang leg. **Hainan** (Hainan population): 1 male, 1 female (SWU), Mingfenggu [鸣凤谷], Mt. Jianfengling [尖峰岭], Ledong County [乐东县], 960–990m, 25.IV.2015, Lu Qiu & Qi-Kun Bai leg.; 3 males (SWU, conserved in 100% alcohol), Mingfenggu, Mt. Jianfengling, Ledong County, 960–990m, 23.IV.2015, Lu Qiu & Qi-Kun Bai leg.; 1 male (SWU), without head and abdomen, Mingfenggu, Mt. Jianfengling, Ledong County, 960–990m, inside a large dead tree hole, beneath the wood dregs, 23.IV.2015, Lu Qiu leg; 3 males (SWU, conserved in 100% alcohol), Mingfenggu, Mt. Jianfengling, Ledong County, 960–990m, 26.IV.2015, Lu Qiu leg.; 1 male (SWU), top of Mt. Jianfengling, Ledong County, 4.V.1983, Mao-Bin Gu leg.; 1 male (SWU), top of Mt. Jianfengling, Ledong County, 9.V.1983, Mao-Bin Gu leg.; 1 male (SWU), top of Mt. Jianfengling, Ledong County, 18.V.1982, Zhi-Qing Chen leg.; 2 males (SWU), Mt. Jianfengling, Ledong County, 16.V.1984, Mao-Bin Gu leg.; 1 female (MHBU), Mt. Bawangling [霸王岭], Changjiang County [昌江县], 8–11.VIII.2006, Ji-Liang Wang & Chao Gao leg. **Taiwan**: 1 male, 1 female (SWU) (*zonata* population), District Shenmu [神木区], Lala Mountain [拉拉山], Taoyuan County [桃源县], Collected and reared by Shih-Chieh Huang; 6 males (LQCC) (*taitoensis* population), Yima Forest Road [依麻林道], Taidong County [台东县], 4.IV.2016, Wen-I Chou leg.

##### Type material examined.

**HOLOTYPE** of *Euthyrrhapha dasytoides*, female (OUM, TYPE ORTH 202), **CHINA: Fujian**: “Amoy (Xiamen) [厦门]”, “E coll. (1830–73)/ W. W. Saunders./ Purchased and pres. ’73 by Mrs. F. W. Hope.”, four determined labels: “TYPE./ WALKER./ Euthyrrapha dasytoides./ Cat. Blatt. B.M. p, 191. 1868.”, “Dasytoides”, “TYPE ORTH: 202/ Euthyrrapha dasytoides Walker/ HOPE DEPT. OXFORD”, “*Eucorydia Aenea* dasytoides Princis, 1963”. **LECTOTYPE** of *Corydia tonkinensis*, female (NHM, #876268), **VIETNAM**: ‘‘Tonkin, Montes Maulon. April, Mai 2–3000, H. Fruhstorfer.”; **PARALECTOTYPE** of *Corydia tonkinensis*, 1 female (NHM, #876269), **VIETNAM**: same data as the lectotype. **HOLOTYPE** of *Eucorydia paucipilos*a, 1 male (SWU, IPP0159), **CHINA: Yunnan**: Tongzi [桐子], Yiliang County [彝良县], Zhaotong City [昭通市], 1050m, 5.VII.1980, Zheng-Jin Luo leg.

##### Diagnosis.

This species resembles *E. pilosa* sp. n., but differs from the latter by: 1) lacking whitish pubescence on the base of tegmina; 2) in male, the hind lateral corners of supra-anal plate more round, cerci longer (Fig. 6A–B); 3) R2 more round (Fig. 6F, G-O), while R2 elongate and rhomboid in *E. pilosa* sp. n. (Fig. 8C). This species also resembles *E. yunnanensis* but is distinctly larger (more than 18 mm including tegmina, while only 15 mm in *E. yunnanensis*), the male has a less concave supra-anal plate, and more round R2, while *E. yunnanensis* has R2 more elongate (Fig. 8B).

##### Redescription.

Male: measurements (mm): body length 11.0–18.5, overall length 18.6–22.3, pronotum length×width: 5.0–5.6×7.3–9.4, tegmen length: 15.1–18.3. Large, metallic bluish green.

Head shining black, slightly metallic blue. Pronotum metallic bluish green to blue, with black setae. Tegmina with basal half metallic bluish green, distal half with a yellow band transversely across the tegmina, sometimes the band interrupted twice and divided into three parts (usually the population from Zhejiang and Fujian, Fig. 5A, D, F, H, J), and sometimes the band interrupted medially and divided into two parts (the population from Taidong, Taiwan, Fig. 4E); the area near the basal edge of the yellow band usually metallic purple, the apical portion of tegmina blackish, slightly metallic purple. Wings hyaline, apex brown, anterior margin of the RA area with an elongate yellow spot, venation brown, median area of M and CuA yellow, some individuals with wings totally dark brown. Legs dark brown to black, slightly pubescent, spines on the legs black, with apex reddish brown.

Abdomen in ventral view, the last sternites black, the rest part orange (usually the populations from Tonkin, Vietnam, and Yunnan, Guangxi, Hainan and Taiwan), sometimes with median blackish widely (usually the populations from Guizhou, Hunan); in dorsal view the last terga black, the rest terga orange, or dark purple in the middle and orange laterally (the populations from Guizhou and Hunan). Supra-anal plate with hind margin slightly obtuse angle concaved, two hind corners slightly round, cerci black, long (Fig. 6A–B). Subgenital plate black, styli robust, black.

Genitalia: L3 slender, strongly curved, apex with a distinct hook (Fig. 6C); appendage sclerite with basal portion roundly protruded toward left, distal portion elongate, apex bud-like (Fig. 6D–E); R2 usually round, left with a shallow protruding and small white part (Fig. 6F and G–O).

Female: body length 12.0–17.5 mm. The coloration and marking pattern are similar to that of male. Abdomen in ventral view with the last two sternites black, the rest of sternites orange, or with median blackish widely (usually the populations from Zhejiang and Fujian).

##### Remarks.

This species is widely distributed from South China to North Vietnam and with distinct geographical variation, which can be divided into six populations. The type population, which is the most widespread, is characterized by the narrow orange band in tegmina and blackish abdomen (Fig. 2; sternites all black and terga all dark purple, but with median three segments orange laterally). This character agrees with that of the type specimen of *Euthyrrhapha dasytoides* Walker. So far this population is known to cover the following localities: South Fujian, East Guizhou and Hunan. Guangdong and Jiangxi, the provinces in between Fujian and Hunan, should also be within this population range, but we did not examine any specimens. The *tonkinensis* population is characterized by the moderate width of orange band, large body size and orange abdomen (Fig. 3; in male, both the sternites and terga with the last 4–5 segments black, the rest of segments totally orange; in female, the last two sternites black and the last five terga black, the remaining segments totally orange). This character is in agreement with that of the type specimen of *Corydia tonkinensis* Kirby. This population is found in Guangxi, Yunnan and North Vietnam. We have examined the type specimen of *Eucorydia paucipilosa* and found it to not be different from the *tonkinensis* population of *E. dasytoides*; thus we treat it as a junior synonym of *E. dasytoides*. The *purpuralis* population is distributed in Zhejiang and Central and North Fujian, which is characterized by the orange band of tegmina twice interrupted and orange abdomen in male but black abdomen in female (Fig. 5; male with the last four segments black, the rest all orange, female with sternites black and terga dark purple, but orange laterally). This population was formerly accepted as an independent species *Eucorydia purpuralis* Hebard, but we found no distinct differences in the male genitalia between *E. purpuralis* and *E. dasytoides*. Thus we here synonymize *E. purpuralis* under *E. dasytoides*. The Hainan population is characterized by the wide orange band in tegmina and orange abdomen (Fig. 4G–J; the scope of the orange area in abdomen larger: sternites and terga with the last three black, the lateral margins of last 4–5 terga black, the rest of segments totally orange). This population is newly discovered from Hainan Island. The *zonata* population is distributed in Taoyuan, Xinzhu (Fuhosho = 内茅埔, Hoozan = 宝山), Gaoxiong (Kosempo = 甲仙, Nanshanchi = 南山溪), Taizhong (Momoyama = 桃山) and Nantou (Horisha/Hori/Polisha = 埔里, Musha = 雾社, Keitao = 溪头, Kwantochi = 关刀溪), Xinbei (Wulai= 乌来), Jiayi (Taihorin = 大蒲林), all in Taiwan (this distribution is based on Asahina, 1971). It was originally treated as an independent species *Eucorydia zonata* Shiraki, but later synonymized under *E. dasytoides* (Princis, 1963). It is similar to the Hainan population; the scope of the orange in the abdomen is larger, but with a narrower orange band in tegmina (Fig. 4A–D). The *taitoensis* population is found in Taidong (= Taito) in south Taiwan. It can be easily recognized by the once interrupted orange band in tegmina (Fig. 4E–F).

The six populations are all well characterized by the features mentioned above. Their male genitalia show only minor differences (see Fig. 6) and we consider them to be the same species. Their distribution is continuous; thus it is not proper to divide them into subspecies. We also found aberrant individuals between some populations, e.g., a male from Yueyang, Hunan is found with the tegmina band twice interrupted, but its abdomen is black (Fig. 2K); two males from the top of Tianmushan, Zhejiang had a black abdomen, but the band was twice interrupted (Fig. 5F–G). These examples also indicate that *E. purpuralis* is conspecific with *E. dasytoides*.

##### Natural history.

At Mingfenggu, Mt. Jianfengling, Hainan in 2015, most individuals were picked up from the ground in the hotel yard by the first author. The individuals on the ground were usually weak or had just died. These species may have inhabited the trees around the hotel and may have fallen down during death, or under the force of wind. A dead cockroach body was found inside the humus in a huge tree bole in the Mingfenggu Forest. A living individual was also observed by the first author on the roof; when approached, it quickly flew away towards the forest. In Taiwan, the male of this species has been observed visiting flowers of *Castanopsis carlesii* and *Acer albopurpurascens* during the day (Wen-I Chou, pers. comm.). On Mt. Tianmushan, males were observed flying through the airflow at the top of Xianrending (De-Yao Zhou, pers. comm.).

##### Distribution.

China: Fujian, Zhejiang, Guizhou, Hunan, Guangxi, Yunnan, Hainan, Taiwan; Vietnam: Tonkin.

#### Eucorydia linglong sp. n.

Taxon classificationAnimaliaBlattodeaCorydiidae

http://zoobank.org/68739643-7855-42BA-90E0-FB7151077EB7

**Figure 7.**
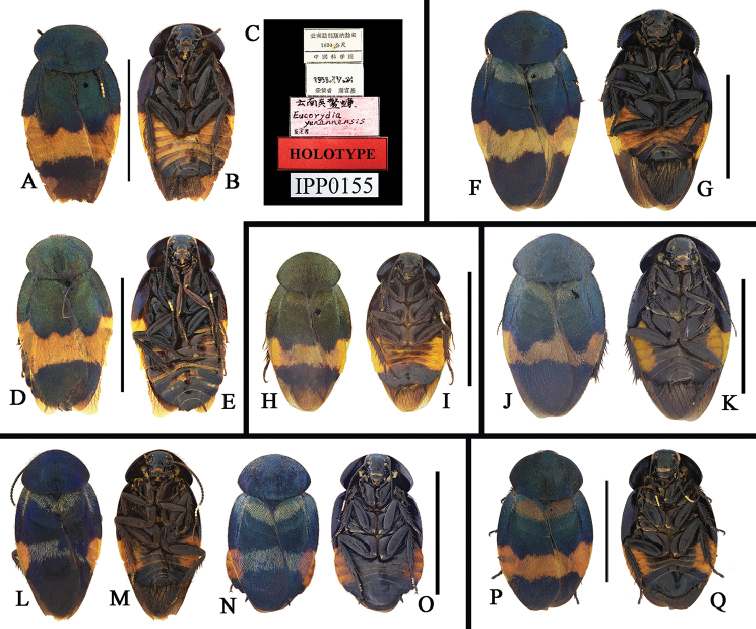
**A–Q** *Eucorydia* species from China **A–E** *E. yunnanensis*: **A–C** holotype, male **D–E** male, from Anshun, Guizhou **F–G** *E. pilosa* sp. n. male holotype from Xiaoheijiang, Yunnan **H–I** *E. guilinensis* sp. n. male holotype from Guilin, Guangxi **J–K** *E. tangi* sp. n. male holotype from Mayanghe, Guizhou **L–O** *E. linglong* sp. n.: **L–M** male holotype from Mt. Jianfengling, Hainan **N–O** female paratype from Shuolong, Guangxi **P–Q** *E.* sp. 2, female from Xiaoheijiang, Yunnan. Scale bars: 1 mm.

**Figure 8.**
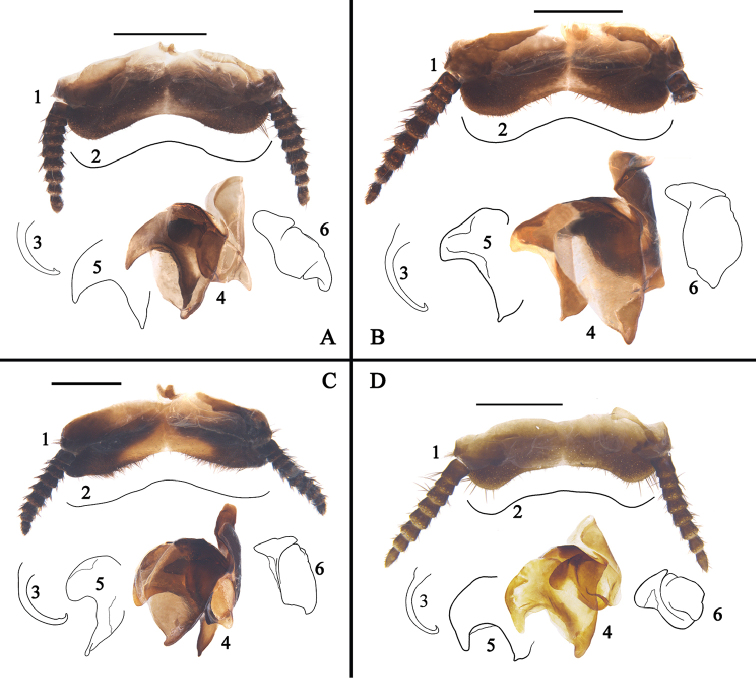
**A–D** Features of *Eucorydia* species (supra-anal plate (**1**), hind margin of supra-anal plate (**2**), genital hook/L3 (**3**), right phallomere with L7 (**4**), appendage sclerite/L7 (**5**), R2 (**6**)) **A** *E. linglong* sp. n. **B** *E. yunnanensis* **C** *E. pilosa* sp. n. **D** *E. guilinensis* sp. n. Scale bars 1 mm.

Figs 7L–O ; 8A ; 14A–D

##### Type material.

**HOLOTYPE: CHINA: Hainan**: male (SWU, A-4666), top of Mt. Jianfengling, Ledong County, 25.IV.1983, Mao-Bin Gu leg. **PARATYPES: CHINA: Hainan**: 1 male (SWU, A-5238), top of Mt. Jianfengling, Ledong County, 9.V.1983, Mao-Bin Gu leg.; 1 male (SWU), Tianchi Lake, Mt. Jianfengling, Ledong County, 8–10.V.1964, Hui Ren leg.; 1 male (SWU, preserved in 100% alcohol), Mt. Limushan, Qiongzhong County, Wuzhishan City, 17.IV.2015, Xin-Ran Li & Zhi-Wei Qiu leg.; **Guizhou**: 1 male (SWU), Xiaoqikong [小七孔], Maolan Natural Reserve, Libo County, Qiangnan Prefecture, 30.V.1998, Jun-Yue Zhi leg.; **Yunnan**: 1 male (SWU), Nasa Town [那洒镇], Guangnan County, Wenshan Prefecture, 1700m, 12.VI.1979, Lin-Bin Lei leg.; **Guangxi**: 1 female (SWU), Shuolong Town [硕龙镇], Daxin County [大新县], Congzuo City [崇左市], 20.V.2016, Yi-Zhou Liu leg.

**Other material examined. CHINA: Guangxi**: 1 male, abdomen missing (MHBU), Jiulong Village [九龙村], Yachang Tree Farm [雅长林场], Leye County, Baise City, 28.VII.2004, Yang Yu & Chao Gao leg.

**Diagnosis.** This species resembles *E. aenea*, *E. forceps*, *E. coerulea*, and *E. gemma* by having two spots on the lateral margins of tegmina, but it can be distinguished from *E. aenea* and *E. forceps* by the arrangement of the white pubescence (band shaped in *E. linglong*, while circle-shaped in *E. aenea* and *E. forceps*); it differs from *E. coerulea* by the white pubescence (with white pubescence in *E. linglong* while without in *E. coerulea*); it can be distinguished from *E. gemma* by the larger body size (more than 10 mm without tegmina in *E. linglong*, while less than 10 mm in *E. gemma*).

##### Description.

Male: measurements (mm): body length 11.0–12.4, overall length 12.5–15.8 (including wings), pronotum length×width 3.3–3.9×5.1–6.0, tegmen length 9.1–11.7. Small, metallic bluish green to deep blue (Fig. 7L–M).

Head shiny, metallic black; antennae (except the whitish segments distally), maxillary palpi and labial palpi brownish black. Pronotum metallic bluish green to deep blue, with black long setae, usually with some white pubescence. Tegmina bluish green to deep blue, blackish toward apex, each lateral margin with an orange spot, tegmina with white pubescence basally, the space between the two orange spots with a strip of white pubescence; wings hyaline, venation brown, distinct. Legs dark brownish black, with brown pubescence, spines on the legs dark brown, with apex reddish brown.

Abdomen in ventral view, the last four sternites (including subgenital plate) dark brown, slightly metallic, the rest with lateral portions orange, median blackish; in dorsal view, the last five terga (including supra-anal plate) black, the rest of terga purplish black but with the three orange lateral portions. Supra-anal plate with hind median roundly concave, lateral hind corners round, cerci short (Fig. 8A); subgenital plate with two long styli.

Genitalia (Fig. 8A): L3 curved, the apex hook with curved part protruded, left base of appendage sclerite with an elongate and sharp process, hind apex with a sharp elongation, R2 elongate, each apex round.

Female: overall length 12.7 mm, similar to male, but with short tegmina which extends only slightly beyond the end of abdomen (Fig. 7N–O).

##### Etymology.

The specific epithet “linglong [玲珑]” means small and elegant in Chinese, in reference to its small and gorgeous body.

##### Remarks.

The female paratype was captured in the karst environment from Guangxi (Fig. 14C–D). This species is also distributed in Vietnam, as seen in a photo taken by Mr. E. Jendek (Fig. 14B); the photo information is: “Cuc-Phuong National Park​, 20°21'58.0"N, 105°35'31.0"E, 27.IV.2012, E. Jendek photoed”. A male individual has also been photographed by Mr. Jin Chen from Manhao [蔓耗镇], Gejiu City [个旧市] in Yunnan (Fig. 14A).

##### Natural history.

A male was found on the ground by accident during a rainy day in Manhao, Yunnan (Fig. 14A, Jin Chen, pers. comm.). The female paratype was collected by Mr. Yi-Zhou Liu from a pile of wood (Fig. 14C).

##### Distribution.

China: Hainan, Yunnan, Guangxi, Guizhou; Vietnam: Hoa Binh.

#### Eucorydia pilosa sp. n.

Taxon classificationAnimaliaBlattodeaCorydiidae

http://zoobank.org/DFF8DDD4-66FF-403C-AC33-E3A839BBBA47

Figs 7F–G ; 8C

Eucorydia dasytoides : Woo et al. 1986: 154 (non E. dasytoides, misidentification).

##### Type material.

**HOLOTYPE: CHINA: Yunnan**: male (SWU), Xiaoheijiang Forest Park [小黑江森林公园], Simao District [思茅区], Pu’er City [普洱市], 25.VII.2009, Zong-Qing Wang leg. **PARATYPES: CHINA: Yunnan**: 1 male (SWU), around Jiujin Township [酒井乡] and Huimin Township [惠民乡], Lancang County [澜沧县], Pu’er City [普洱市], 1135m, 10.VI.1980, no collector recorded; 1 male (SWU), Mengzhe Town [勐遮镇], Menghai County [勐海县], Xishuangbanna Prefecture [西双版纳州], 870m, 1.VI.1958, Zhi-Zi Chen leg.; 1 male (SWU), Mengzhe Town, Menghai County, Xishuangbanna Prefecture, 875m, 4.VII.1958, Fu-Ji Pu leg.

##### Other material examined.

**CHINA: Yunnan**: 1 male (SWU), abdomen missing, around Jiujin Township and Huimin Township, Lancang County, Pu’er City, 1135m, 10.VI.1980, no collector recorded.

##### Diagnosis.

This species resembles the *tonkinensis* population of *E. dasytoides*, but differs from the latter by: 1) having whitish pubescence at the base of tegmina, 2) the cerci of male is shorter; 3) median of supra-anal plate widely “V” shaped (Fig. 8C, No.1-2) (the concave part narrow, more round laterally in the supra-anal plate of *tonkinensis* population of *E. dasytoides*); 4) R2 elongate and rhomboid (Fig. 8C, No. 6) (nearly round in the *tonkinensis* population of *E. dasytoides* (Fig. 6F, G–O, No. 2).

##### Description.

Male: measurements (mm): body length 13.8–15.1, overall length 18.9–21.5, pronotum length×width 4.5–4.8×6.8–7.4, tegmen length 14.1–15.9. Median size, metallic purplish blue (Fig. 7F–G).

Head shiny, metallic blackish blue; antennae (except the whitish segments), maxillary palpi and labial palpi black. Pronotum metallic blue, with black long setae. Tegmina with basal half metallic blue, the basal half of anal areas with white pubescence, distal half with a yellow band transversely across the tegmina which occupies nearly 1/6 of the tegmen length, the basal edge of the band metallic purple, the apical portion of tegmina blackish, slightly metallic purple. Wing brown, venation brown, distinct, median of the outer margin with elongate yellow spots, a yellow trail originates from each spot and extends to the median of the wing. Legs black, slightly pubescent, spines on the legs black, with apex reddish brown.

Abdomen in ventral view, margins pubescent, the last two and the lateral portions of 6^th^ to 7^th^ sternites black, slightly metallic, the rest orange. Supra-anal plate with hind median broadly concave, cerci short (Fig. 8C, No. 1-2); subgenital plate with two long and robust styli.

Genitalia (Fig. 8C): L3 with the apex hook gently curved; the appendage sclerite with basal left roundly protruding, hind portion elongate, gradually narrower; R2 elongate, slightly rhomboid shaped, details as in Fig. 8C, No. 6.

Female unknown.

##### Etymology.

The species epithet “pilosa” refers to its whitish pubescence on tegmina base.

##### Remarks.

This species and *E. dasytoides* were confused and misidentified in Woo et al. (1986). They treated this species as *E. dasytoides*, and an *E. dasytoides* individual from Yunnan as a new species *E. paucipilosa* Woo, Guo & Feng.

##### Natural history.

The holotype was found inside a mixture of humus by the second author.

##### Distribution.

China: Yunnan.

#### Eucorydia hilaris

Taxon classificationAnimaliaBlattodeaCorydiidae

(Kirby, 1903), new record to China

Figs 9D–F ; 14L–N

Corydia hilaris Kirby, 1903: 406 (based on 1 male and 2 females, locality unknown); Kirby 1904: 167 (catalogue).
Eucorydia hilaris : Hebard 1929: 98; Princis 1957: 90 (designated the male as lectotype and the 2 females as paralectotypes); Princis 1963: 82.

##### Type material examined.

**LECTOTYPE** of *Corydia hilaris*, male (NHM, #877092), no data recorded.

##### Diagnosis.

**Figure 9.**
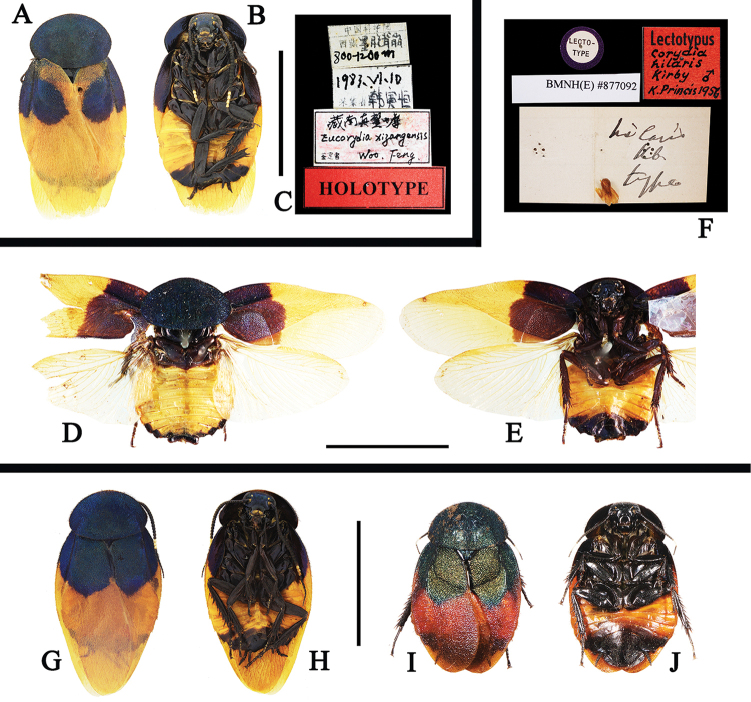
**A–J** Habitus of *Eucorydia* species **A–C** male holotype of *E. xizangensis* **D–F** male holotype of *E. hilaris* [D–F photographed by Zong-Qing Wang, copyright The Natural History Museum, United Kingdom (NHM)] **G–J** *E. splendida* sp. n.: **G–H** male holotype **I–J** female paratype [**I–J** photographed by Jia-Zhi Zhang]. Scale bars 10 mm.

Male: head metallic black. Pronotum metallic blue. Tegmina in resting position with basal half metallic blue, the distal half totally yellow; the border between the two colors nearly straight in the middle, median area of the metallic part more protruded than the lateral areas. Wings yellow. Legs brown. Abdomen orange both in dorsal and ventral view; in ventral view, S7, S8 and lateral portions of S6 brown; in dorsal view, lateral margins of T6-T8 brownish black narrowly, T9 brownish black. Supra-anal plate brownish black, hind margin concave, obtuse angle-shaped; subgenital plate brownish black (Fig. 9D–E).

This species resembles *E. splendida* sp. n. and *E. xizangensis*, but differs from both by the border shape between the orange part and metallic part on tegmina. Also, its supra-anal plate with hind margin concave at an obtuse angle, while supra-anal plate with hind margin roundly concave in *E. splendida* and straight in *E. xizangensis*.

##### Remarks.

This species is described based on three specimens without collection data. Two photographs of this species were obtained from Yunnan, China (Fig. 14L–M), but no specimens are available for study. The photo information are listed as followed: Fig. 14L, one male, Yakou [垭口], Huanglianshan [黄莲山], Lvchun County [绿春县], Honghe Prefecture [红河州], Yunnan, photographed by Jian-Yun Wang; Fig. 14M, one female with ootheca, South Ximeng County [西盟县], Pu’er City [普洱市], Yunnan, 1000 m, Dong Lin leg., photographed by Chao Li.

##### Natural history.

The female (Fig. 14M) was found behind a tree; it was lying on the dead part of the tree when captured (Fig. 14N, Chao Li, pers. comm.).

##### Distribution.

China: South Yunnan.

#### Eucorydia xizangensis

Taxon classificationAnimaliaBlattodeaCorydiidae

Woo & Feng, 1988

Figs 9A–C ; 10A

Eucorydia xizhangensis Woo & Feng, 1988: 29, fig.1, male holotype.

##### Type material examined.

**HOLOTYPE** of *Eucorydia xizhangensis*, male (SWU, IPP0156), **CHINA: Tibet**: Beibeng Township [背崩乡], Motuo County [墨脱县], Nyingchi City [林芝市], 800–1200m, 10.VI.1983, Yin-Heng Han leg.

##### Diagnosis.

**Figure 10.**
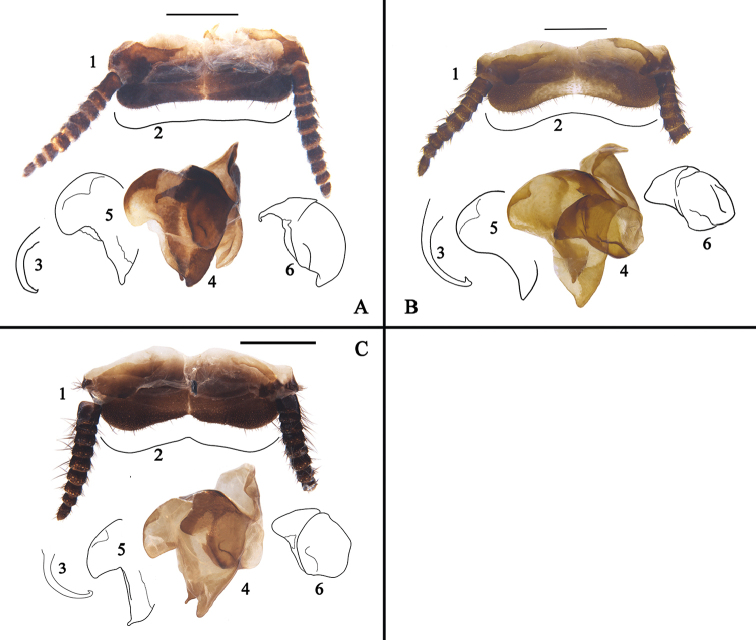
**A–C** Features of *Eucorydia* species (supra-anal plate (**1**), hind margin of supra-anal plate (**2**), genital hook/L3 (**3**), right phallomere with L7 (**4**), appendage sclerite/L7 (**5**), R2 (**6**)) **A** *E. xizangensis* **B** *E. splendida* sp. n. **C** *E. tangi* sp. n. Scale bars 1 mm.

This species resembles *E. splendida* sp. n. and *E. hilaris*, but can be easily distinguished by its tegmina marking pattern, the basal metallic portions of *E. xizangensis* are much reduced, while the latter two have the basal portion of tegmina widely metallic bluish green. *E. xizangensis* can also be easily distinguished from *E. splendida* by: 1) hind margin of supra-anal plate straight (Fig. 10A, No. 1-2), while concave in *E. splendida* (Fig. 10B, No. 1-2); 2) L3 with apex less hooked (Fig. 10A, No. 3), while L3 strong hooked in *E. splendida* (Fig. 10B, No. 3); 2) R2 with an elongate irregular process on the left, apex of the process curved (Fig. 10A, No. 6), while R2 normally protruded toward left in *E. splendida* (Fig. 10B, No. 6).

##### Redescription.

Holotype (male): measurements (mm): body length 14.1, overall length 16.5, pronotum length×width 4.5×6.6, tegmen length 12.4. Median size, deep metallic blue with large yellow area (Fig. 9A, B).

Head metallic bluish black, ocelli distinct. Pronotum deep metallic blue, margins with black setae. Mesonotum and metanotum purplish brown. Tegmina orange, Sc field of each tegmen metallic bluish purple, which areas extend and direct inward, forming a large round spot around CuP in cfr; wings yellow entire. Legs shiny black, with black pubescence, spines on the legs black.

Abdomen in ventral view orange, the last four sternites metallic black, styli black, S6 slightly yellowish medially and with two small yellow spots laterally; in dorsal view orange, T5 and T6 with lateral margins narrowly blackish brown, T7 and T8 with median orange, lateral parts black. Supra-anal plate black, hind margin nearly straight, cerci black, long (Fig. 10A, No.1-2); subgenital plate with robust styli.

Genitalia (Fig. 10A): L3 slender, strongly curved, apex thinner; appendage sclerite narrow, base enlarged, round, left portion roundly protruded, distal portion elongate, apex bud-like protruded; R2 with an elongate irregular process on the left, apex of the process curved.

Female unknown.

##### Distribution.

China: Southeast Tibet.

#### Eucorydia splendida sp. n.

Taxon classificationAnimaliaBlattodeaCorydiidae

http://zoobank.org/B34013FA-CEF2-43F4-90C9-C59711B8E402

Figs 9G–J ; 10B

##### Type material.

**HOLOTYPE: CHINA: Yunnan**: male (IZCAS), Laowo Township [老窝乡], Lushui County [泸水县], Nujiang Prefecture [怒江州], 1670m, 25.VI.1981, Shu-Yong Wang leg. **PARATYPE: CHINA: Yunnan**: 1 female (JZZC), Baihualing [百花岭], Mt. Gaoligong [高黎贡山], Baoshan City [保山市], 25.VII.2016, no collector recorded.

##### Diagnosis.

See under *E. hilaris* and *E. xizangensis*.

##### Description.

Male: measurements (mm): body length 13.0, overall length 16.2, pronotum length×width 3.8×6.3, tegmen length 13.2. Median size, metallic bluish green with large yellow area (Fig. 9G–H).

Head metallic bluish black, ocelli small but distinct. Pronotum metallic bluish green, with black setae. Mesonotum and metanotum purplish brown. Tegmina in resting position with basal half metallic bluish green, distal half orange entire, the border between the two colors W-shaped, the distal border of the metallic area much more blue than the rest of area; wings totally yellow. Legs brownish black, with black pubescence, spines on the legs brownish black, with apical portion brown.

Abdomen in ventral view bright orange, the last three sternites dark shiny brown, S6 with lateral parts brown, median orange; in dorsal view, bright orange, T6 and T7 with lateral margins narrowly blackish brown, T8 and T9 with median orange, lateral parts black. Supra-anal plate black, hind margin roundly concave, lateral corners round, cerci black (Fig. 10B, No. 1-2); subgenital plate black, styli robust, dark brown.

Genitalia (Fig. 10B): L3 slender, strongly curved, apical portion with a distinct hook, the apex of the curved point of the hook protruding; appendage sclerite with left portion roundly enlarged at the base, distal portion gradually narrowed, apex angular; R2 with left slightly protruded, shape as in Fig. 10B, No. 6, R3 with anterior portion reflexed.

Female: Coloration pattern similar to male, tegmina distinctly shorter than that of male (Fig. 9I–J).

##### Etymology.

The specific epithet “splendida” refers to its bright appearance.

##### Distribution.

China: West Yunnan.

#### Eucorydia yunnanensis

Taxon classificationAnimaliaBlattodeaCorydiidae

Woo, Guo & Feng, 1986

Figs 7A–E ; 8B

Eucorydia yunnanensis Woo, Guo & Feng, 1986: 155, figs 7–8; Feng et al. 1997: 175, fig. 70a–b.

##### Material examined.

1 male (SWU), **CHINA: Guizhou**: Anshui City [安顺市], 1000m, V.1982, Ping-Zhang Feng leg.

##### Type material.

**HOLOTYPE** of *Eucorydia yunnanensis*, male (SWU, IPP0155), **CHINA: Yunnan**: “Mengsong, Banna, Menglong, Yunnan” (Now Mengsong Township [勐宋乡], Menghai County [勐海县], Xishuangbanna Prefecture [西双版纳州]), 1600m, 24.IV.1958, Fu-Ji Pu leg.

##### Diagnosis.

*Eucorydia yunnanensis* superficially resembles *E. guilinensis* sp. n., but the former can be distinguished from the latter by the following characters: 1) without any whitish pubescence on pronotum and tegmina (Fig. 7A, D), while mixed with many yellowish white pubescence on pronotum and the base of tegmina in *E. guilineneis* (Fig. 7H); 2) the orange band on tegmina is quite wide (Fig. 7A, D), while narrow in *E. guilineneis* (Fig. 7H); 3) hind margin of the concave part in supra-anal palte narrow (Fig. 8B, No. 1-2), while wide in *E. guilineneis* (Fig. 8D, No. 1-2); appendage sclerite less curved than that of *E. guilineneis* (Fig. 8B, No. 5 and 8 D, No. 5); R2 elongate, the left basal portion with a long protrusion (Fig. 8B, No. 6), while R2 short and round, and left basal portion with a short protrusion (Fig. 8D, No. 6).

##### Redescription.

Male: measurements (mm): body length 12.7–13.0, overall length 15.2–15.5, pronotum length×width 3.8-4.1×6.0-6.3, tegmen length 11.9–12.4. Small size, metallic bluish green, with yellow band (Fig. 7A–B, D–E).

Head black, slightly metallic blue, ocelli small but distinct. Pronotum metallic bluish green to green, with black setae. Tegmina in resting position metallic bluish green to green, distal portion with a wide yellow band, apical portion bluish brown, slightly metallic; wings brown, median yellowish. Legs brownish black, with black pubescent, spines on the legs brownish black, with apical portion reddish brown.

Abdomen in ventral view orange, the last three sternites black, S6 with lateral parts brown, median orange; in dorsal view orange, T6 and T7 with lateral margins narrowly blackish brown. Supra-anal plate black, hind margin with median deeply concave, lateral hind corners round, cerci black, long (Fig. 8B, No. 1-2); subgenital plate black, styli robust.

Genitalia (Fig. 8B): L3 slender, strongly curved, apical portion with a distinct hook; appendage sclerite with left portion strongly protruded at the base, distal portion with apex bud-like; R2 elongate, basal left with a long protruding, shapes as in Fig. 8B, No. 6, R3 with anterior portion reflexed.

Female unknown.

##### Distribution.

China: Yunnan, Guizhou (new record).

#### Eucorydia guilinensis sp. n.

Taxon classificationAnimaliaBlattodeaCorydiidae

http://zoobank.org/FB5C4253-3D89-4212-804C-E3423B08C89A

Figs 7H–I ; 8D

##### Type material.

**HOLOTYPE: CHINA: Guangxi**: male (IZCAS), Yanshan [雁山], Guilin City [桂林市], 200m, 17.V.1963, Shu-Yong Wang leg. **PARATYPES: CHINA: Guangxi**: 9 males (IZCAS), same data as the holotype; 1 male (IZCAS), Yanshan, Guilin City, 200m, 12.VII.1963, Shu-Yong Wang leg.; 3 males (IZCAS), Yanshan, Guilin City, 200m, 16.V.1963, Yong-Shan Shi leg.; 1 male (IZCAS), Yanshan, Guilin City, 200m, 19.V.1963, Yong-Shan Shi leg.

##### Diagnosis.

See under *Eucorydia yunnanensis*.

##### Description.

Male: Measurements (mm): body length 10.7–11.4, overall length 13.8–14.2, pronotum length×width 3.4–3.6×5.3–5.5, tegmen length 11.0–11.3. Small size, generally metallic green with orange band (Fig. 7H–I).

Head black, slightly dark metallic green, vertex with black pubescence and mingled with some yellowish white pubescence. Pronotum metallic green, with distinct short yellowish white pubescence, and mingled with some long black setae. Tegmina metallic green, in resting position, the distal half with a transverse orange band, occupying approximately 1/5 of the tegmen length, apical portion of the tegmen purplish brown, slightly metallic; surface of the basal portion of the tegmina and the orange band with short yellowish white pubescence. Wing transparent, slightly brownish, RA area with a slender yellow spot, RP area brown, venation brown. Legs dark brown, with brown pubescence, spines on the legs yellowish brown.

Abdomen in ventral view, the last four sternites dark brown, the rest of sternites orange, with median slightly brownish; in dorsal view, T8 and T9 and lateral parts of T6 and T7 dark brown, slightly metallic, the rest of terga orange. Supra-anal plate dark brown, hind margin widely concave, cerci dark brown (Fig. 8D, No. 1-2); subgenital plate dark brown, with robust styli.

Genitalia (Fig. 8D): L3 slender, strongly curved, apex with a distinct hook (Fig. 8D (3)); the appendage sclerite with anterior strongly protruded and curved toward left-posterior, distal portion with a small bud-like process (Fig. 8D); R2 small and round, left with a short protruding and a white area, shape as in Fig. 8D.

Female unknown.

##### Etymology.

Specific epithet indicates the type locality: Guilin City, in Guangxi.

##### Distribution.

China: Guangxi.

#### Eucorydia tangi sp. n.

Taxon classificationAnimaliaBlattodeaCorydiidae

http://zoobank.org/13AC255F-3D2D-4132-BFFF-838D32D3B1FF

Figs 7J–K ; 10C

##### Type material.

**HOLOTYPE: CHINA: Guizhou**: Male (SWU, ex SHNU), Lijiaba [李家坝], Mayanghe Natural Reserve [麻阳河自然保护区], Yanhe County [沿河县], 700m, 7.VI.2007, Liang Tang leg. **PARATYPES: CHINA: Guizhou**: 5 males (GZU), Daheba [大河坝], Yanhe County [沿河县], 450–700m, 5–12.VI.2007, Qiong-Zhang Song leg.

##### Diagnosis.

This species resembles *Eucorydia guilinensis* sp. n., but can be distinguished from the latter by the following characters: 1) body larger (15.9–16.3 mm) and broader (8.6–8.8 mm), while body small (13.8–14.2 mm) and narrow (7.0–7.2 mm) in *E. guilinensis*; 2) the white pubescence on tegmina is mainly limited to the suture of left tegmen and 1/3 of basal anal area on both tegmina, while the pubescence more widely distributed at the base of tegmina in *E. guilinensis*; 3) the concave part of supra-anal plate more straight (Fig. 10C, No. 1-2), while more round in *E. guilinensis* (Fig. 8D, No. 1-2); 4) the appendage sclerite with basal left protruded, distal margin of the protruded part straight (Fig. 10C, No. 5), while the appendage sclerite with basal left protruded and strongely curved toward posterior (Fig. 8D, No. 5).

##### Description.

Male: Measurements (mm): body length 12.7–13.2, overall length 15.9–16.3, pronotum length×width 3.9–4.1×6.8–7.0, tegmen length 12.6–12.8. Median size, broad, generally metallic green with orange band (Fig. 7J–K).

Head metallic greenish black, vertex with black pubescence. Pronotum metallic greenish blue entire, with short white pubescence, margins with black setae. Tegmen short and broad, metallic greenish blue, in resting position, the distal half of tegmina with a transverse orange band, occupying nearly 1/7 of the tegmen length, apical portion of the tegmen metallic bluish brown; surface of the basal edges of the tegmina, the sutural margin of left tegmen, and the orange band covered with short white pubescence. Wing transparent, slightly brownish. Legs dark brown, with blackish pubescence, spines on the legs brown.

Abdomen in ventral view brownish black, lateral margins orange except the last four sternites; in dorsal view, terga purplish black, T3-T5 orange laterally. Supra-anal plate dark brown, hind margin concave, cerci dark brown, short (Fig. 10C, No. 1-2); subgenital plate with robust styli.

Genitalia (Fig. 10C): L3 slender, curved, apex with a distinct hook; the appendage sclerite with basal left protruding, distal margin of the protruded part straight, distal portion with a bud-like process; R2 round, basal left with a shallow protruding, shape as in Fig. 10C, No. 6.

Female unknown.

##### Etymology.

This species is named in honor of Mr. Liang Tang (SHNU), the collector of the holotype.

##### Distribution.

China: Guizhou.

### Species outside China

#### Eucorydia aenea

Taxon classificationAnimaliaBlattodeaCorydiidae

(Brunner von Wattenwyl, 1865)

Fig. 11A–G

Corydia aenea Brunner von Wattenwyl, 1865: 340, male, “Indes orientales”; Walker 1869: 126; Brunner von Wattenwyl 1893: 39, t. 1, f. 15, female; Kirby 1904: 167 (Corydia aenea); Shelford 1906: 504; Hanitsch 1927: 41; Princis 1950: 203.
Eucorydia aenea : Hebard 1929: 98; Princis 1963: 81; Asahina 1971: 258 (part).

##### Type material examined.

**LECTOTYPE** of *Corydia aenea*, **here designated**, male (NHMV), **INDIA**: “Ostindien, fr. Fieber (handwriting), Coll. Br. v. W. (East India, Fieber leg., collection of Brunner von Wattenwyl)”, with a blue label: “6128”, a red label with unrecognized letter, a designated label: “LECTOTYPE/ *Corydia aenea* Brunner von Wattenwyl, 1865 ♂/ des. Clyde Qiu (= Lu Qiu) 2016”.

##### Other material examined.

**INDIA**: 2 males (NHMV), same data as the lectotype, one with a round yellow labeled: “18”. **VIETNAM**: 1 male (NHMV), “Cochinchina (Currently in South Vietnam), Saussure, Coll. Br. v. W.”, a bluish green label: “8494”; 2 males (GMNH), all with the following labels: “♂/ Cochinchine/ M??? Saussure”, a yellow determination label: “*Corydia aenea* Br. ♀”; **MYANMAR**: 1♀ (NHMV), “Palon/ (Pegù) / L. Fea. VIII.IX.87”, “Collectio Br. v. W.”, a blue label: “19.084.”; 2♀♀ (GMNH), all with the following labels: “Palon/ (Pegù)/ L. Fea. VIII.IX.87”, “620/ 8g/ Birmania./ Indesor./ Mus. de Genes”, two determination labels: “*Corydia aenea* Br. ♀” (yellow label), “*Corydia aenea*”; **THAILAND**: 1 male (NHM, No. #876266), “Thailand/ LOT. 2522 S. LOT/ Sam Ngow, Tak./ June. 12. 1959/ ? COLL”, a label with number: “105”, “Pres by Com Inst Ent/ B M 1964-2”, “*Eucorydia aenea* (Brunner)/ det. J. A. Meadows, 1968”.

##### Diagnosis.

Total length (including tegmina) nearly 15.0 mm for male, 12.0 mm for female. Body metallic blue, head metallic blue, legs dark brown, tegmina metallic blue, anterior lateral margin of the tegmen with an elongate yellow spot, tegmen surface with circle white pubescence. Abdomen in ventral view brownish black, three segments orange laterally, in dorsal view dark with metallic blue, three segments orange laterally.

**Figure 11.**
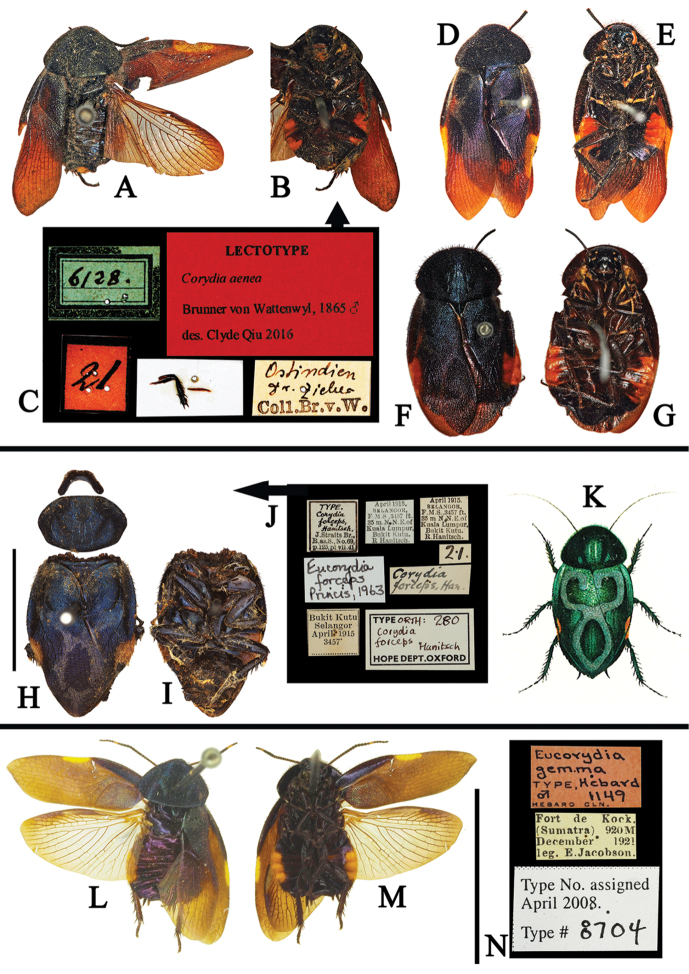
**A–N** *Eucorydia species* outside China **A–G** *E. aenea*: **A–C** lectotype of *Corydia aenea*, male **D–E** male, from east India **F–G** female, from Palon, Myanmar [A–G © by Natural History Museum Vienna, NOaS Image Collection, Harald Bruckner, published with permission] **H–K** *E. forceps*: **H–J** holotype of *Corydia forceps*, male [H–J photographed by Katherine Child and provided by Amoret Spooner, copyright Oxford University Museum of Natural History, Oxford (OUM)] **K** original figure of *Corydia forceps* in Hanitsch (1915) **L–N** holotype of *Eucorydia gemma*, male [**L–N** provided by Jason Weintraub, copyright by the Academy of Natural Sciences of Drexel University, Philadelphia, United States (ANSP)]. Scale bars 10 mm, **A–G, K** without scale bar.

##### Remarks.

This species is characterized by tegmina with two elongate yellow spots, whitish pubescence, and the abdomen with lateral portions orange emarginated. The original description didn’t mention how many specimens were designated as types. We found three specimens from NHMV which were all labeled “Ostindien, fr. Fieber, Coll. Br. v. W.”. None had a type label, except one with a red label which was handwritten with an illegible letter. We consider this specimen as the one which Brunner von Wattenwyl studied (Brunner von Wattenwyl 1865); it is the only one to possess an opened tegmen and wing, and the original description paid much attention to the tegmen and wing. Also, because of the red label, we designated it as the lectotype.

Walker (1868) reported *Eucorydia dasytoides* var. β from Siam as “abdomen beneath with a large luteous patch on each side. Fore wings without a band, but with an elongated spot on the costa”. This description and locality agree with *E. aenea*, so we consider this *E. dasytoides* var. β as *E. aenea*.

##### Distribution.

East India, Thailand, South Vietnam, Myanmar.

#### Eucorydia forceps

Taxon classificationAnimaliaBlattodeaCorydiidae

(Hanitsch, 1915)

Fig. 11H–K

Corydia forceps Hanitsch, 1915: 125 (male holotype, “Bukit Kutu, Selangor, 3000m”), Plate 7, fig. 41; Hanitsch 1923: 466.
Eucorydia forceps : Hebard 1929: 14; Bruijning 1948: 149 (1 male, “Aur Kumanis, Sumatra”, 1 female, “Belang”, all in Leiden Museum); Princis 1963: 83.

##### Type material examined.

**HOLOTYPE** of *Corydia forceps*, ♂ (OUM, TYPE ORTH 280), **MALAYSIA**: two identical labels: “April 1915. SELANGOR, F. M. S., 3457 ft, 35 m N. N. E. of Kuala Lumpur, Bukit Kutu. R. Hanitsch.”, “Bukit Kutu/ Selangor/ April 1915/ 3457”, one number label: “21”, four determination labels: “TYPE./ *Corydia forceps*, Hanitsch./ J. Straits Br., B, as.S., No.69, p.125, pl.vii.41.”, “*Corydia forceps*, Han.”, “TYPE ORTH: 280/ *Corydia forceps* Hanitsch/ HOPE DEPT. OXFORD”, “*Eucorydia forceps* Princis, 1963”.

##### Diagnosis.

Male: Total length about 15 mm, body metallic blue, legs and abdomen metallic blue. Tegmina with two small yellow spots laterally. Tegmina covered with white pubescence, the arrangement as in Fig. 11K. Lateral portions of abdomen orangish emarginated.

This species resembles *E. aenea*, but with legs and abdomen metallic blue, and the yellow spots on tegmina are small.

##### Distribution.

Malaysia: Kuala Lumpur.

#### Eucorydia gemma

Taxon classificationAnimaliaBlattodeaCorydiidae

Hebard, 1929

Fig. 11L–N

Eucorydia gemma Hebard, 1929: 98 (male holotype, “Fort de Kock, Sumatra”); Hanitsch 1929: 288 (6 examples, “Fort de Kock, 920m”), with a comparison with E. coerulea; Bruijning 1948: 149 (2 males and 1 sex unknown from Amsterdam Museum, “Fort de Kock, Sumatra”); Princis 1953: 203 (1 female, from Java); Princis 1963: 82.

##### Type material examined.

HOLOTYPE of *Eucorydia gemma*, male (ANSP, #8704), **INDONESIA**: “Fort de Kock. (Sumatra) 920M/ December. 1921/ leg. E. Jacobson.”, a red label: “*Eucorydia gemma* TYPE, Hebard ♂ 1147/ HEBARD CLN.”, “Type No. assigned April 2008./ Type # 8704”.

##### Other material examined.

1 female (NRM), **INDONESIA**: Java, with determination label “*Eucorydia gemma* Heb., ♀, K. Princis det, 1952”

##### Diagnosis.

Consulting the former descriptions (Hebard 1929; Hanitsch 1929; Bruijning 1948) and the holotype re-examined, this species is characterized as follows: body length 8.0–8.7 mm, overall length 10.4–11 mm, tegmen length 8.0–8.5 mm, pronotum length×width 2.9–3.0×3.9–4.2 mm. Small size, metallic bluish green, tegmen with a narrow orange streak on the costal margin, basal third of anal area with greyish white pubescence, right tegmen where covered by the left shiny purple, mesonotum, metanotum and 1–4 abdominal terga purple, the rest black, lateral borders of 3-5 abdominal sternites orange, abdomen shiny black in ventral view, lateral margins of segments 4 to 6 orange.

##### Remarks.

Besides the type locality, Princis (1953) recorded one female from Java as *E. gemma*.

##### Distribution.

So far this species is recorded from the type locality Fort de Kock, Sumatra, and Java.

#### Eucorydia coerulea

Taxon classificationAnimaliaBlattodeaCorydiidae

(Shelford, 1906)

Fig. 12A–C

Corydia coerulea Shelford, 1906: 272 (male holotype, “Mt. Matang, 3000 feet”); Hanitsch 1915: 125; Hanitsch 1923: 466.
Eucorydia coerulea : Hebard 1929: 14; Bruijning 1948: 41, 149; Princis 1963: 81.

##### Type material examined.

**HOLOTYPE** of *Corydia coerulea*, male (OUM, TYPE ORTH 203), **MALAYSIA**: two same labels: “N. W. BORNEO, Sarawak, about 3500 ft., Mt. Matang, nr. Kuching. Coll. *June. 00.* Pres. 1905 by the Sarawak Museum.”; “TYPE. ♂. R. SHELFORD, *Corydia Coerulea*. T. E. S., Lon., 1906, p. 272-3.”; “Matang, 3600/ June 189 1900”; “*Eucorydia coerulea* Princis, 1963”; “1905/ 599”; “TYPE ORTH: *Corydia coerulea* Shelford./ HOPE DEPT. OXFORD”.

##### Diagnosis.

Male overall length 13.5 mm, tegmen length 10 mm (Shelford, 1906). Body in dorsal view brilliant metallic blue. Head slightly metallic blue. Tegmina metallic blue, with some obscure orange spots on the disc (Shelford, 1906), each lateral margin of tegmina with an elongate, narrow yellow spot. Wings blackish, hyaline. Mesonotum and metanotum purplish brown. Legs slightly metallic blue. Abdomen orange both in dorsal and ventral view, in dorsal view, the last three segments metallic blue, subgenital plate bright metallic blue.

This species is similar to *E. aenea*, but its tegmina has no white pubescence, the last three segments of abdomen are much more metallic than those of *E. aenea*, and the remaining segments of abdomen are totally orange while *E. aenea* has the median of the abdomen blackish.

**Figure 12.**
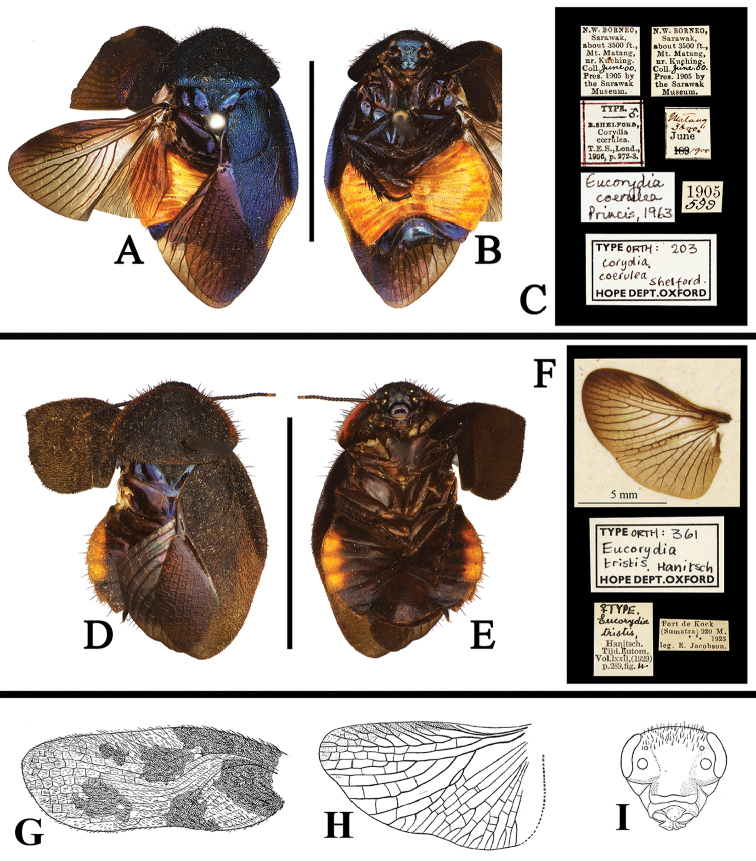
**A–I** *Eucorydia species* outside China **A–C** holotype of *Corydia coerulea*, male **D–F** holotype of *Eucorydia tristis*, female [A–F photographed by Katherine Child and provided by Amoret Spooner, copyright Oxford University Museum of Natural History, Oxford (OUM)] **G–I** original figures of *Eucorydia multimaculata* in Bruijning (1948), male: **G** tegmen **H** wing **I** head. Scale bars 10 mm.

##### Distribution.

Malaysia: Sarawak.

#### Eucorydia multimaculata

Taxon classificationAnimaliaBlattodeaCorydiidae

Bruijning, 1948

Fig. 12G–I

Eucorydia multimaculata Bruijning, 1948: 150 (male holotype, “Siantar, Sumatra”, in Amsterdam Museum), fig. 55a-c; Princis 1963: 83.

##### Material examined.

None.

##### Diagnosis.

Based on Bruijning (1948): male body length 9.5 mm, pronotum length×width 3.0×4.5 mm, tegmen length 9.4 mm. Head blackish brown, pronotum metallic green with orangish hairs. Tegmina yellow, basal portions metallic green, median and distal portions with six irregular patches, shape as in Fig. 12G, abdomen blackish brown, with three sternites orange laterally.

This species can be easily recognized by its multiple peculiar color patches on tegmina.

##### Distribution.

Indonesia: Sumatra.

#### Eucorydia tristis

Taxon classificationAnimaliaBlattodeaCorydiidae

Hanitsch, 1929

Fig. 12D–F

Eucorydia tristis Hanitsch, 1929: 289 (female holotype, “Fort de Kock, 920m”), Fig. 4, left wing from holotype; Bruijning 1948: 149; Princis 1963: 83.

##### Type material examined.

**HOLOTYPE** of *Eucorydia tristis*, female (OUM, TYPE ORTH 361), **INDONESIA**: “Fort de Kock (Sumatra) 920 M./ 1925/ leg. E. Jacobson.”; “♀ TYPE./ *Eucorydia tristis*, (handwritten)/ Hanitsch. Tijd. Eutom. Vol. lxxii, (1929)/ p.289, fig. 4.”; “TYPE ORTH: 361/ *Eucorydia tristis*. Hanitsch (handwritten)/ HOPE DEPT. OXFORD”.

##### Diagnosis.

Female: overall length: 9.5 mm, body length 9.0 mm, pronotum length×width 3.0×4.2 mm, tegmina length 6.5 mm. Body brownish black with yellowish pubescence, margins of body sparsely covered with long and rough blackish setae. Pronotum dull black, with apex and lateral margins narrowly dull reddish. Tegmina dull black entire, covered with small yellow pubescence. In dorsal view, mesonotum and metanotum metallic blue, lateral borders of abdomen with 3-5 segments orange both in dorsal and ventral view.

This species is distinguished by its dull blackish coloration, which makes it unique in *Eucorydia*.

##### Distribution.

Indonesia: Sumatra.

#### Eucorydia westwoodi

Taxon classificationAnimaliaBlattodeaCorydiidae

(Gerstaecker, 1861)

Corydia westwoodi Gerstaecker, 1861: 114 (female holotype, Assam); Brunner von Wattenwyl 1865: 339 (French translation of the original description); Walker 1869: 126; Kirby 1904: 167; Hanitsch 1927: 41.
Eucorydia westwoodi : Hebard 1929: 96 (designed it as type species of Eucorydia); Hanitsch 1932: 80 (synonymized E. maxwelli under E. westwoodi); Bruijning 1948: 149; Princis 1963: 83.
Eucorydia plagiata : Asahina 1971: 259 (2 males, eastern Nepal, misidentification).

##### Material examined.

None.

##### Diagnosis.

Combining the original description (Gerstaecker 1861) and the French description (translated from the original description) (Brunner von Wattenwyl 1865), this species is characterized as follows: female length 13.0 mm; antennae black; pronotum dull blue, margins with long black hair; tegmina orange, each tegmen with a blackish blue strip originates from the anterior margin, extends from the base to the middle and curved inwardly, ends bulbously, an oval spot of the same color is situated at the sutural margin of the tegmina and ends in front of the middle, one third of the apical portion of the tegmina brownish black.

This species resembles *E. ornata* by its tegmina marking pattern, but differs from the latter by the pronotum, the former with pronotum unicolored, while the latter has two yellow elongate spots on the pronotum laterally.

##### Remarks.

This species was reported from Assam by one single female (Gerstaecker, 1861). Hebard (1929) established the genus *Eucorydia* and designated this species as the type species. Asahina (1971) recorded two males of *E. plagiata* from Nepal (one from “East Nepal”, the other from “Dharan, at Grkha camp”); from the description, we consider them as *E. westwoodi* because their pronotum lacks yellow spots. In addition to the examples Asahina examined, he also mentioned one specimen without yellow spots from Darjeeling.

##### Distribution.

India: Assam (type locality), Darjeeling (Asahina 1971); Nepal (Asahina 1971).

#### Eucorydia ornata

Taxon classificationAnimaliaBlattodeaCorydiidae

(Saussure, 1864)

Figs 13A–G ; 14J–K

Melestora ornata Saussure, 1864: 341 (“India, Bombay”); Walker 1868: 60.
Corydia ornata : Saussure 1869: 280; Walker 1870: 9; Kirby 1904: 167.
Eucorydia ornata : Hebard 1929: 98; Princis 1963: 83.
Corydia plagiata Walker, 1868: 58; Kirby 1904: 167; Hanitsch 1927: 41. **Syn. n.**
Eucorydia plagiata : Princis 1950: 203; Princis 1957: 90; Princis 1963: 83.
Corydia elegans Brunner von Wattenwyl, 1893: 39 (“Carin Chebà (900-1100 m.)”).

##### Type materials examined.

**HOLOTYPE** of *Corydia ornata*, male (GMNH), **INDIA**: “Indes orient./ M.H de Saussure/ ♂”, “*Corydia westwoodi* Gers/ Indes./ var.”, “*Corydia ornata* ♂/ Sauss.”, also with a description script, all information is in handwriting. **LECTOTYPE** of *Corydia plagiata*, female (NHM, No. #876270), a round label: “48/ 22”, a round blue emarginated label: “LECTO-TYPE”, a red label: “Lectotypus/ *Corydia plagiata* Walker ♀ (handwritten)/ K. Princis 1956 (handwritten)”, “plagiata (handwritten)”.

##### Diagnosis.

Male overall length nearly 15.0 mm (including tegmina), female overall length about 11.5 mm. Pronotum black, slightly dark bluish, each lateral border with one elongate yellow spot. Tegmina yellow, with pattern similar to that of *E. westwoodi*; lateral margin of tegmen with an elongate blackish brown stripe that originates from the base and extends to the middle of tegmen margin; the apical portion curves inward and apex becomes bulbous, the distal portion of anal area with an large blackish oval spot, apex of tegmina brownish. Some individual with the yellow areas of tegmina reduced, and occupied by large blackish brown markings. Legs brown to dark brown, slightly bluish, abdomen yellow, with apical portion brown.

**Figure 13.**
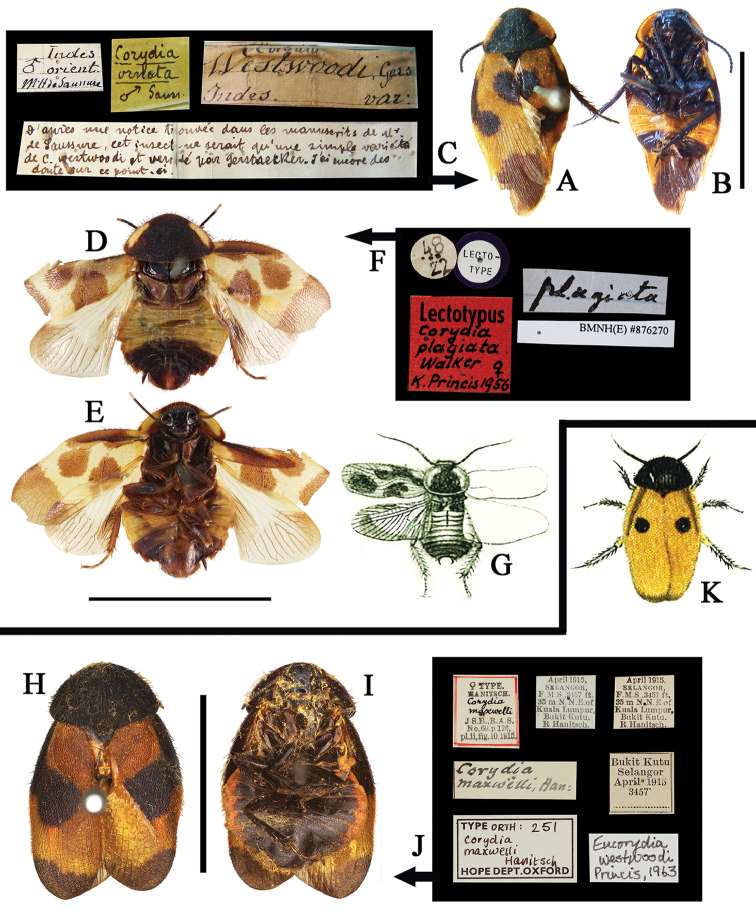
**A–K** *Eucorydia* species outside China **A–G** *E. plagiata*: **A–C** holotype of *Corydia ornata*, male [A–C photographed by Peter Schwendinger, copyright Museum of Natural History, Geneva, Switzerland (GMNH)] **D–F** lectotype of *Corydia plagiata*, female [D–F photographed by Zong-Qing Wang, copyright by The Natural History Museum, United Kingdom (NHM)] **G** original figure of *Corydia elegans* in Brunner von Wattenwyl (1893) **H–K** *E. maxwelli*: **H–J** type of *Corydia maxwelli*, female **K** original figure of male *Corydia maxwelli* in Hanitsch (1915) [**H–J** photographed by Katherine Child and provided by Amoret Spooner, copyright Oxford University Museum of Natural History, Oxford (OUM)]. Scale bars 10 mm.

**Figure 14.**
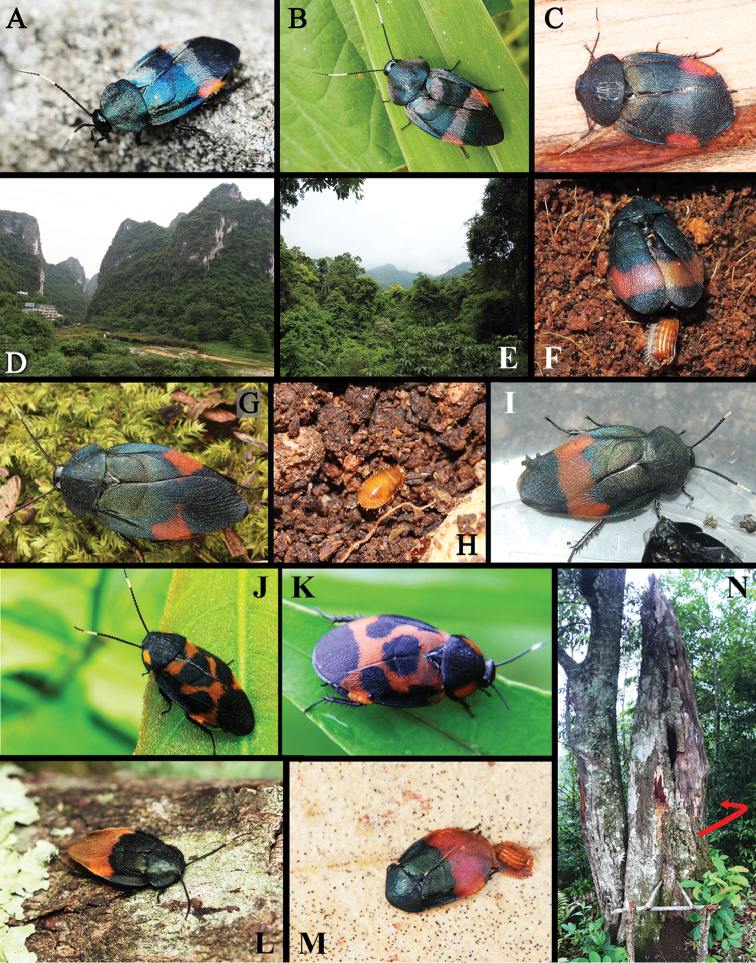
**A–D** habitats of *E. linglong* sp. n.: **A** male, Manhao, Yunnan **B** male, Cuc-Phuong National Park​, Vietnam **C** female, Shuolong, Guangxi **D** environment in Shuolong, Guangxi **E–I** habitats of *E. dasytoides*: **E** habitat in Mingfenggu, Mt. Jianfengling, Hainan **F** a female with ootheca under lab conditions **G** a male from Taiwan **H** a nymph under lab conditions **I** a newly captured male from Mt. Jianfengling **J–K** *E. ornata* from India: **J** male from Madhya Pradesh **K** female from Maharashtra **L–N** habitats of *E. hilaris*: **L** male from Lvchun, Yunnan **M–N** newly-collected female and the tree where captured, Pu’er, Yunnan [Photograph **A** by Jin Chen; **B** by Eduard Jendek; **C** by Ye Liu; **D** by Yi-Zhou Liu; **G** by Dash Huang; **J–K** by Aniruddha Dhamorikar; **L** by Jian-Yun Wang; **M–N** by Chao Li; the rest by Lu Qiu].

##### Remarks.

This species was originally recorded from Bombay, India (Saussure 1864). Brunner von Wattenwyl (1893) described *E. elegans* from Carin Chebà, Myanmar, but this was later synonymized under *E. ornata* by Kirby (1904). We did not examine the type specimens of *E. elegans*, but the original description and illustration of *E. elegans* are in agreement with the type specimen of *E. plagiata*; thus we consider Kirby’s synonymy (1904) reasonable. After examining the type of *E. ornata* and *E. plagiata*, we found the two types to have the same marking pattern, and the pronotum all with two elongate spots on the borders; thus we treat *E. plagiata* as a junior synonym of *E. ornata*.

From the original description of *E. westwoodi*, we found *E. ornata* quite similar to *E. westwoodi* by its tegmina marking. Yet they exhibit a distinct difference on the pronotum. The former has its pronotum with yellow elongate spots laterally, while the latter has a unicolored pronotum. *E. ornata* may be one variation of *E. westwoodi*, or a subspecies. We temporarily maintain them as two species since current knowledge is too limited to solve the problem.

##### Distribution.

India: Bombay; Myanmar.

#### Eucorydia maxwelli

Taxon classificationAnimaliaBlattodeaCorydiidae

(Hanitsch, 1915) stat. rev.

Fig. 13H–K

Corydia maxwelli Hanitsch, 1915: 126 (2 males respectively from Maxwell’s Hill, Perak and Lingga Mt., Sarawak; 2 females from Bukit Kutu, Selangor); Hanitsch 1923: 466.
Eucorydia maxwelli : Hebard 1929: 14.
Eucorydia westwoodi : Hanitsch 1932: 80 (1 male from Siboga, Sumatra); Bruijning 1948: 149; Princis 1963: 83.

##### Type material examined.

**TYPE** of *Corydia maxwelli*, female (OUM, TYPE ORTH 251), two identical labels: “April 1915./ SELANGOR, F. M. S., 3457 ft, 35 m N. N. E. of Kuala Lumpur, Bukit Kutu./ R. Hanitsch.”; “Bukit Kutu/ Selangor/ April 1915/ 3457”; “♀ TYPE/ HANITSCH./ *Corydia maxwelli* (handwritten)/ J.S.B., R.A.S./ No. 69. p 126, pl.ii, fig.10.1915.”; “*Corydia maxwelli*, Han: (handwritten)”; “*Eucorydia westwoodi* Princis, 1963 (handwritten)”; “TYPE ORTH: 251/ *Corydia maxwelli* Hanitsch/ HOPE DEPT. OXFORD”.

##### Diagnosis.

This species may show sexual dimorphism. Male overall length about 11.0 mm (including tegmina); head orange, pronotum black; tegmina orange, each tegmen with a round black spot in the center, and with apex blackish; coxae, femora and abdomen orange, tibiae, tarsi and cerci black. Female overall length about 11.0 mm; head, pronotum, legs and cerci black, tegmina orange; lateral of tegmen with a stripe occupying the entire half of basal margin, and curved inward near the median, the curved part slightly enlarged and quadrate, the entire anal areas are encircled by the two stripes, apex of tegmina blackish; abdomen black, lateral margins orange.

##### Remarks.

Hanitsch (1915) described *Corydia maxwelli* from southeast Asia. He realized this species showed sexual dimorphism, and later synonymized it under *E. westwoodi* (Hanitsch, 1932) based on the female tegmina pattern. However, this was in error: *E. westwoodi* indeed is similar to *E. maxwelli* by the lateral blackish strips and the blackish apex on tegmina; but the former had an additional large oval spot near the anal area, while the later lacks a spot near the anal area. In addition, *E. westwoodi* is distributed in North India and Nepal, while *E. maxwelli* is in southeast Asia. Since the two species are geographically disjunct we believe they are unlikely to be the same species, and we suggest restoring the status of *E. maxwelli*.

The sexual dimorphism of this species is unusual in *Eucorydia*. Other *Eucorydia* species show weak sexual dimorphism; the female generally resembles the male, but with shorter tegmina and wings. Hanitsch (1915) at first has stated that the female he described may be another species. Later, he correctly treated it as sexual dimorphism and synonymized *C. maxwelli* under *E. westwoodi* (Hanitsch, 1932), but without convincing reasons. According to the collection data in Hanitsch (1915), the males and females are not from the same place, which could lead to false pairing. We consider the female more likely to be a separate species.

##### Distribution.

Malay Peninsula; Sumatra and Borneo.

#### Eucorydia yasumatsui

Taxon classificationAnimaliaBlattodeaCorydiidae

Asahina, 1971

Eucorydia yasumatsui Asahina, 1971: 256 (♂ holotype, “Omotodake, Ishigaki Island”, 2♂♂ paratypes and 1 nymph, “Iriomoto Island”), figs 1–2, 9; Asahina 1991: 55, fig. 35, Plate 3, fig. 10, holotype, one paratype and one nymph; Fujita and Machida 2014: 193.

##### Material examined.

None.

##### Diagnosis.

Male: small, body length 10.0 mm, tegmen length 10.0-11.0 mm (Asahina 1971); pronotum and tegmina unicolored, metallic blue, legs black, abdomen brownish black but with lateral margins yellow. Female is slightly larger than male, abdomen blackish brown, lateral margins yellow, or abdomen yellow, apex slightly brownish (see Fujita and Machida 2014).

##### Remarks.

To date, this is the only *Eucorydia* species recorded in Japan. However, Sakamaki and Tsuda (2006) also recorded one unknown species from Uji Island. That species superficially resembles the *purpuralis* population of *E. dasytoides* but whether it is a new species is still undetermined.

##### Distribution.

Japan: Ishigaki Island and Iriomoto Island.

### Unnamed species

#### Eucorydia

Taxon classificationAnimaliaBlattodeaCorydiidae

sp. 1

Eucorydia aenea : Asahina 1971: 258, (1 male and 1 female, “Doi Pui, 1685m, N. Thailand”) (misidentification).

##### Material examined.

None.

##### Remarks.

This species was reported in Asahina (1971), where it was misidentified as *E. aenea*. According to Asahina, it is characterized as following: male body length 14.5 mm, tegmen length 13.0 mm; female body length 14.0 mm, tegmen length 9.0 mm. Female similar to the male, head shiny black, pronotum metallic blue. Tegmina metallic blue, basal half of the anal areas yellow, median of the tegmina with three large yellow spots, the lateral two on the margins, slightly elongate, the median one transverse. Abdomen orange in dorsal view, terga 1, 7 and 8 black laterally, terga 9, supra-anal plate and cerci black, subgenital plate and styli black.

After examining the specimens of *E. aenea* that Brunner v. W. studied, this species shows a very different tegmina color pattern from *E. aenea*. *Eucorydia* sp. 1 has more yellow spots on its tegmina (two yellow spots at base and three large yellow spots distributed in the middle), while *E. aenea* only has two elongate yellow spots on the tegmina margins. The marking pattern of the tegmina also distinctly differentiates it from other congeners. Thus it is considered an unnamed species, but due to lack of specimens for study, it is simply recorded here for future study.

##### Distribution.

Thailand: Chiang Mai (Doi Pui).

#### Eucorydia

Taxon classificationAnimaliaBlattodeaCorydiidae

sp. 2

Fig. 7P–Q

##### Material examined.

**CHINA: Yunnan**: 1 female (SWU), Xiaoheijiang Forest Park, Simao District, Pu’er City, 24.VII.2009, Zong-Qing Wang leg.

##### Remarks.

This species resembles *E.* sp. 1 with the basal half of anal areas yellow on tegmina, but differs from the latter by the orange band on distal half of tegmina, while the latter with three large yellow spots on distal half of tegmina. The last two sternites of this species are metallic bluish black, the remaining sternites are brownish black in the middle and orange laterally. The tegmina pattern of this species can be easily distinguished from the other species of this genus, which indicates it could be a new species. However, because no male specimen is available to us, we record it here for future study.

##### Distribution.

China: Yunnan (Pu’er).

## Supplementary Material

XML Treatment for Eucorydia

XML Treatment for Eucorydia dasytoides

XML Treatment for Eucorydia linglong

XML Treatment for Eucorydia pilosa

XML Treatment for Eucorydia hilaris

XML Treatment for Eucorydia xizangensis

XML Treatment for Eucorydia splendida

XML Treatment for Eucorydia yunnanensis

XML Treatment for Eucorydia guilinensis

XML Treatment for Eucorydia tangi

XML Treatment for Eucorydia aenea

XML Treatment for Eucorydia forceps

XML Treatment for Eucorydia gemma

XML Treatment for Eucorydia coerulea

XML Treatment for Eucorydia multimaculata

XML Treatment for Eucorydia tristis

XML Treatment for Eucorydia westwoodi

XML Treatment for Eucorydia ornata

XML Treatment for Eucorydia maxwelli

XML Treatment for Eucorydia yasumatsui

XML Treatment for Eucorydia

XML Treatment for Eucorydia
